# Foster mothers’ attachment styles and children’s attachment security in middle childhood: a mixed-methods study of the Turkish foster family service model

**DOI:** 10.3389/fpsyg.2026.1986177

**Published:** 2026-10-12

**Authors:** Cezmi Ervüz, Özlem Karakuş

**Affiliations:** 1 Child Protection and Care Services Program, Department of Child Care and Youth Services, Artova Vocational School, Tokat Gaziosmanpaşa University, Tokat, Türkiye; 2 Department of Social Work, Faculty of Health Sciences, Selçuk University, Konya, Türkiye

**Keywords:** attachment security, birth family contact, child welfare, foster care, intergenerational transmission of attachment, middle childhood, mixed methods, Türkiye

## Abstract

Attachment theory predicts that a caregiver’s own attachment organization shapes the security of the child in their care, but the evidence comes almost entirely from biological parent–infant dyads. Whether it extends to a caregiving relationship with no shared genetic history, no shared infancy, and a competing attachment figure still present is largely untested, particularly in middle childhood, when children can report on the relationship themselves. This study examined whether the attachment security of children aged 9–12 in foster care is associated with their foster mother’s attachment style, with the age at which they entered protection, and with the frequency of contact with their birth family, and explored how foster mothers and children describe those relationships. In this explanatory sequential mixed-methods study, 314 foster mother–child dyads across eight Turkish provinces, 13.2% of the eligible national population, completed the Relationship Scales Questionnaire and the Kerns Security Scale. In-depth interviews with 10 foster mothers sampled across all four attachment classifications, and their 10 children, were analysed thematically. Children’s security differed by foster mother attachment style, *F*(3, 310) = 13.16, *p* < 0.001, η^2^ = 0.113, 90% CI [0.058, 0.164]; children of securely attached foster mothers scored higher than children of fearful (d = 0.83), preoccupied (d = 0.76) and dismissing (d = 0.78) foster mothers. Security was also higher among children placed at or before age three, *F*(2, 311) = 4.34, *p* = 0.014, η^2^ = 0.027, and differed by contact frequency, *F*(4, 309) = 2.56, *p* = 0.039, η^2^ = 0.032, although this effect did not survive Bonferroni correction and the pattern was non-monotonic rather than linear: children with no contact scored as high as those with monthly contact. No exploratory comparison survived correction. Four foster mother themes and three child themes converged with this pattern, and the children’s metaphors, safe harbour, guide and light, protective shield, and loving from the heart, described a functioning secure base. The caregiver’s own attachment organization was associated with several times more variance in children’s security than either placement history or contact frequency, which supports attachment-informed assessment and support of foster carers.

## 1 Introduction

Attachment theory holds that repeated experience with a caregiver who is available and responsive under stress consolidates into an internal working model, a set of expectations about whether closeness is worth seeking and whether distress will be met (Bowlby, 1958; Ainsworth, 1979). Why that representation matters for the child is well established. Meta-analytic evidence links attachment security in childhood to greater social competence with peers (Groh et al., 2014), and attachment insecurity to internalizing symptoms across childhood and adolescence (Brumariu and Kerns, 2010) and to the development of anxiety (Kerns and Brumariu, 2014). Middle childhood is the period in which this representation becomes reportable: the child holds an articulated set of expectations about whether the caregiver is available and whether distress will be met, and those expectations organise how the child seeks help, regulates distress and approaches peers. The security of the attachment representation in this period is therefore not only a description of one relationship but a marker of the child’s wider socioemotional functioning, which is what makes the question of how it is formed consequential.

One of the most productive claims in developmental psychology is that these models are transmitted: a caregiver’s own state of mind with respect to attachment predicts the security of the child in their care. van IJzendoorn (1995) established a large association between adult attachment representations and infant attachment classification while showing that caregiver sensitivity accounted for only part of it, the residual that became known as the transmission gap; Verhage et al. (2016) synthesised 95 samples and confirmed that the association holds, though more modestly than once assumed.

Almost all of that evidence comes from biological parent–infant dyads, where the transmission claim is confounded with shared genes, shared prenatal environment, and continuous caregiving from birth. Foster care removes each of those: foster mother and child share no genetic history, the relationship begins after infancy in most placements, the child arrives carrying a working model built elsewhere, and in many cases a birth parent remains an active figure. If transmission reflects how a caregiver’s own representation organizes her caregiving conduct rather than biology or continuity, it should be observable here. If it does not appear here, the phenomenon is narrower than the field believes.

The available evidence suggests that it does appear, but the base is thin and concentrated in infancy. Dozier et al. (2001) found concordance between foster mothers’ attachment state of mind and the attachment quality of infants placed with them, approaching the magnitude seen in biological dyads, and Stovall-McClough and Dozier (2004) showed that foster infants’ attachment behaviour organized around the new caregiver within two months of placement along a trajectory that depended on her state of mind. Bernard et al. (2012) demonstrated experimentally that an attachment-based intervention with caregivers reduced disorganized attachment among maltreated children, establishing that the caregiver side of the dyad is causally implicated rather than merely correlated, and Dozier and Lindhiem (2006) showed that foster parents differ markedly in commitment to the child, a construct distinct from sensitivity and specific to non-biological caregiving. Schofield and Beek (2005, 2009) drew this material into the secure base model of long-term foster care, in which availability, sensitivity, acceptance, cooperation and family membership are the dimensions through which foster carers build security.

The children who enter this relationship do not arrive as blank slates. Children in out-of-home care show elevated rates of insecure and, in particular, disorganized attachment: a recent systematic review found insecure and disorganized patterns predominating among children in residential care (Martínez-Usarralde et al., 2025), Jorjadze et al. (2023) found German foster children lower in behavioural security and higher in representational disorganization than controls, Dalgaard et al. (2025) found Danish foster children elevated specifically on disorganization, and McWey (2004), in the study that most directly models attachment styles among foster children, located the problem in the child’s history rather than the placement. Against this, Kliewer-Neumann et al. (2023) followed newly placed foster children across their first year and found security rising to normative levels within six months. Foster care is a period of potential reorganization, and the question is what determines its direction.

Two features of the placement itself are usually nominated. The first is the age at which the child enters care: Smyke et al. (2010) found in the Bucharest Early Intervention Project that earlier placement in foster care produced better attachment outcomes among institutionalised children, and Bernier et al. (2004) and Spieker et al. (2014) reported converging evidence that the early window matters. The second is contact with the birth family, where the literature is less settled than policy discourse assumes. Systematic and scoping reviews conclude that the effects of visitation on child adjustment are inconsistent, and that contact quality and parental sensitivity predict adjustment better than frequency does (Poitras et al., 2022a; Ruiz-Romero et al., 2022); more frequent contact combined with lower parental sensitivity predicts greater externalizing behaviour (Poitras et al., 2022b). Work from the Málaga group has made the mechanism concrete: foster families, social workers, and birth families all attribute post-visit distress to poor planning, unsuitable venues, and inadequate supervision rather than to contact itself (García-Martín et al., 2018; Fuentes et al., 2018). Visit quality can be measured observationally (García-Martín et al., 2024), and it is modifiable through structured support of birth parents (González-Pasarín et al., 2023; Bernedo and González-Pasarín, 2025a, 2025b). Children, foster parents, and social workers evaluate the same visits very differently (Salas Martínez et al., 2016), and kinship placements show distinctive contact patterns (León et al., 2017). The convergent implication is that frequency is a poor proxy for what matters.

Two further gaps shape the present study. First, this literature is overwhelmingly about infants and preschoolers. Middle childhood is a period in which attachment reorganizes around availability and open communication rather than proximity, and in which children can report on the relationship in their own words (Kerns et al., 1996; Kerns and Brumariu, 2014). The Kerns Security Scale was developed for this developmental window and has meta-analytic evidence of validity (Brumariu et al., 2018). Yet, it has rarely been used with foster children alongside a measure of the caregiver’s own attachment. Second, the evidence base is almost entirely from Western Europe and North America. Türkiye offers an informative case: the foster family service model (*koruyucu aile*) expanded rapidly after 2012 as part of a deliberate de-institutionalization policy, so that by 2024, approximately 10,084 children were living with foster families, while 14,509 remained in institutional care (Ministry of Family and Social Services, 2024). It operates in a context of strong extended-family obligation and religiously framed care of orphans, and is delivered through a state agency that screens, approves, and monitors foster families and regulates contact with birth parents (Akan Ellis, 2021; Karaveli and Erükçü Akbaş, 2026; Kelebek and Kucur, 2025).

The gap is therefore precise. Transmission is well established in biological dyads and partially established for foster infants, and essentially untested for foster children in middle childhood, where the child’s own report is available and where placement history and continuing birth-family contact are simultaneously in play. Studies of foster children’s security typically measure either the child or the caregiver, rarely both in the same dyad, and rarely alongside qualitative accounts from both parties; where they do combine them, the strands are usually reported side by side rather than integrated, which forfeits the main advantage of the design.

This study addresses that gap with a dyadic, multi-informant, explanatory sequential mixed-methods design in a national sample. Its contribution is twofold. Empirically, it shows that a foster mother’s attachment style is associated with several times more variance in a school-aged foster child’s attachment security than either age at placement or contact frequency, in a relationship with no biological or infancy continuity, and that the association between contact frequency and security is non-monotonic rather than linear. Theoretically, it extends the transmission-of-attachment framework beyond biological dyads and beyond infancy. It uses foster mothers’ and children’s own accounts to show what transmission looks like from inside the relationship.

Four research questions follow from this framework. The first three are directional and confirmatory, derived from the transmission and secure base models; the fourth is exploratory.

*RQ1:* Does the attachment security of children aged 9–12 in the foster family service model differ according to the age at which they were taken into protection?

*RQ2:* Does their attachment security differ according to the frequency of contact with their birth family?

*RQ3:* Does their attachment security differ depending on their foster mother's attachment style?

*RQ4:* How do foster mothers and children in the foster family service model describe their attachment experiences, and how do those accounts relate to the quantitative pattern?

## 2 Materials and methods

### 2.1 Design

An explanatory sequential mixed-methods design was used (Creswell and Plano Clark, 2011; Fetters et al., 2013). A cross-sectional quantitative phase (Karasar, 2011) established the pattern of association between foster mothers’ attachment styles, placement variables, and children’s attachment security. A qualitative phase was then designed from those results: foster mothers were purposively selected so that all four attachment classifications were represented, and the child in her care was also interviewed, using a phenomenological approach focused on how individuals describe the events they live through (Creswell, 2002; Patton, 2014). The strands were integrated at interpretation through a joint display (Guetterman et al., 2015; Bazeley, 2024). The qualitative strand is reported in accordance with COREQ (Tong et al., 2007); the checklist is provided as Supplementary Material.

### 2.2 Setting: the Turkish foster family service model

Because the setting will be unfamiliar to many readers, it is described briefly. In Türkiye, children who cannot remain with their birth families are taken into protection by court order and placed by the Ministry of Family and Social Services, which operates institutional care, adoption, and the foster family service model. Foster families apply to provincial directorates, are assessed through home visits and social investigations conducted by professional social workers, and are approved, trained, and monitored by a dedicated foster care unit. Approved families receive a monthly maintenance payment and ongoing supervision. Every placement is assigned a caseworker: a professional social worker employed by the Ministry and based in the provincial directorate’s foster care unit, who carries out the home visits, monitors the placement and holds the child’s administrative file. Caseworkers are staff of the child protection authority and were not members of the research team.

The model has three forms: *temporary* care (*geçici koruyucu aile*) for short crisis placements, *kinship or close-environment* care (*akraba veya yakın çevre koruyucu aile*) with relatives or adults already known to the child, and *long-term* care (*süreli koruyucu aile*), the predominant form, for placements expected to continue until majority. Legal guardianship remains with the state, the placement does not sever the child’s legal relationship with the birth family, and contact with birth parents is arranged, scheduled and in most cases supervised by the provincial directorate rather than negotiated privately between the two families.

### 2.3 Participants and sampling

#### 2.3.1 Quantitative strand

According to Ministry statistics, 2,379 children aged 9–12 and 2,085 foster mothers were in the foster family service model nationally, the target population. An accessible population of 682 children was defined across eight provinces selected for geographic diversity and number of children in the model: İstanbul (192), İzmir (107), Ankara (86), Konya (73), Gaziantep (64), Samsun (65), Kahramanmaraş (61) and Malatya (34).

The required sample size was calculated with the finite-population formula n = (N × t^2^ × p × q) ÷ (d^2^(N − 1) + t^2^ × p × q), with N = 682, t = 1.96, p = q = 0.50 and d = 0.05, giving a minimum of 246 dyads. Quota sampling allocated this minimum across the eight provinces in proportion to their population share, and quotas were filled by convenience sampling through the caseworkers of the provincial foster care units, the only personnel with lawful access to family contact details. The realised sample of 314 dyads exceeded every provincial quota (Supplementary Table S1) and represents 13.2% of the eligible national population in this age band. A sensitivity analysis indicates 80% power to detect η^2^ ≥ 0.034 (*f* = 0.19) at α = 0.05 with four groups; achieved power was 1.00 for RQ3, 0.75 for RQ1 and 0.73 for RQ2.

Inclusion criteria were: aged 9–12 at the time of data collection; resident with the foster family for at least 12 months; and able to read, write, understand and speak Turkish without difficulty. Exclusion criteria were: age outside the 9–12 band; fewer than 12 months in the current placement; difficulty in reading, writing, understanding or speaking Turkish sufficient to prevent independent completion of the child instrument; and, at the matching stage, any dyad for which one or more of the three instruments was missing or incomplete.

Only foster mothers were surveyed, by design. In the Turkish model the foster mother is in practice the registered primary caregiver in the great majority of placements; the Kerns Security Scale was administered with respect to the mother figure, so mother–child dyads are the comparable unit; and including fathers would have required a father-referenced administration that the fieldwork protocol did not permit. The exclusion of foster fathers restricts the scope of the findings and is addressed in the Limitations.

Three hundred and fourteen children aged 9–12 (M = 10.61, SD = 1.13) participated, 57.3% of them girls. Two fifths (40.1%) had entered protection at age three or younger and a fifth (20.7%) at seven or older; the most common reason for entry was neglect and/or abuse (43.6%). Just under half (49.7%) had no contact with their birth family, 12.4% had monthly contact and 17.5% contact every three months. Most (72.6%) had biological siblings, and most (72.6%) were in long-term foster care, with 17.5% in kinship or close-environment care and 9.9% in temporary care.

The 314 foster mothers were aged 26 to 65 (M = 49.68, SD = 7.62). Most were married (92.4%), had biological children of their own (58.9%), were not in paid employment (67.8%), and had primary or lower secondary education (60.8%). Full sociodemographic detail for both groups is given in Table 1.

**Table 1:** Sociodemographic characteristics of the quantitative sample (*N* = 314 dyads).

Variable	Category	*n*	%
Panel A: children			
Sex	Male	134	42.7
	Female	180	57.3
Age at entry into protection	3 years or younger	126	40.1
	4–6 years	123	39.2
	7 years or older	65	20.7
Reason for entry into protection	Neglect and/or abuse	137	43.6
	Parental divorce	42	13.4
	Abandoned or found without a guardian	39	12.4
	Parental intellectual disability	35	11.1
	Birth outside marriage	31	9.9
	Parental death	30	9.6
Duration in the foster placement	3 years or less	86	27.4
	4–6 years	126	40.1
	7 years or more	102	32.5
Frequency of contact with birth family	No contact	156	49.7
	Monthly	39	12.4
	Every three months	55	17.5
	Every six months	31	9.9
	Once a year	33	10.5
Biological siblings	Yes	228	72.6
	No	86	27.4
Number of biological siblings	None	86	27.4
	One	76	24.2
	Two or three	106	33.8
	Four or more	46	14.6
Type of foster care service	Long-term	228	72.6
	Kinship/close environment	55	17.5
	Temporary	31	9.9
Panel B: foster mothers			
Marital status	Married	290	92.4
	Single	24	7.6
Duration of marriage (of those married)	7 years or less	209	72.1
	8 years or more	81	27.9
Manner of marriage (of those married)	Family introduction	194	66.9
	Own acquaintance	96	33.1
Biological children	Yes	185	58.9
	No	129	41.1
Education	Primary	148	47.1
	Lower secondary	43	13.7
	Upper secondary	69	22.0
	University	54	17.2
Employment	Not employed	213	67.8
	Employed	101	32.2
Self-rated family relationships	Good	259	82.5
	Other	55	17.5

Children’s age M = 10.61, SD = 1.13 (range 9–12). Foster mothers’ age M = 49.68, SD = 7.62 (range 26–65).

#### 2.3.2 Qualitative strand

Ten foster mothers and the ten children in their care were recruited through the caseworkers of the provincial directorates. Selection was purposive: following the quantitative analysis, foster mothers were identified whose Relationship Scales Questionnaire profile placed them in each of the four attachment classifications, and volunteers were sought from each group. The realised qualitative sample comprised four foster mothers classified as secure, three as preoccupied, two as fearful, and one as dismissing. All ten children were placed with these ten foster mothers, so each interview pair represents one dyad.

Sample size was determined by information power rather than data saturation (Malterud et al., 2016): the aim was narrow, the sample highly specific to the phenomenon, the analysis theory-supported, dialogue quality strengthened by piloting, and the strategy cross-case. The ten foster mothers were aged 39 to 64 and had been fostering for three to eleven years; six were in long-term and four in kinship placements. The ten children were aged 9 to 12 and had entered protection between the ages of one and ten. Full characteristics of each dyad are given in Table 2.

**Table 2:** Characteristics of the ten foster mother–child dyads in the qualitative strand.

#	Foster mother: age, education, employment	Marital status; biological children	Years fostering	Service type	RSQ classification	Child: age, sex	Age at entry into protection	Prior institutional care	Birth-family contact
1	64, primary, not employed	Married; 3	9	Kinship	Fearful	12, M	3	Yes	Father only
2	60, primary, not employed	Married; 2	10	Long-term	Preoccupied	12, F	1	Yes	None
3	57, lower secondary, not employed	Married; 2	11	Long-term	Preoccupied	11, F	1	Yes	None
4	39, primary, not employed	Married; 3	6	Kinship	Secure	10, M	4	Yes	None
5	39, university, employed	Married; 1	8	Kinship	Secure	12, F	10	Yes	None
6	44, university, employed	Single; 0	4	Kinship	Dismissing	12, M	1	Yes	None
7	51, primary, not employed	Married; 0	6	Long-term	Fearful	9, M	2	No	None
8	44, university, employed	Married; 1	6	Long-term	Secure	12, F	3	Yes	None
9	45, upper secondary, not employed	Married; 4	3	Long-term	Preoccupied	12, F	8	No	None
10	50, primary, not employed	Married; not recorded	6	Long-term	Secure	10, M	4	No	None

RSQ, Relationship Scales Questionnaire. Foster mothers are referred to in the text as FM1–FM10 and children as C1–C10, corresponding to the dyad numbers above.

### 2.4 Measures

#### 2.4.1 Personal information form

A form developed by the first author recorded the foster mother’s age, marital status, education, employment, income and number of biological children, and the child’s age, sex, age at and reason for entry into protection, duration in the placement, type of foster care service, biological siblings, their care status and frequency of contact with them, frequency of contact with the birth family, and the birth family’s place of residence. It was completed by the caseworker responsible for the child and family, which reduced reliance on carer recall for administrative variables.

#### 2.4.2 Relationship scales questionnaire (RSQ)

Foster mothers’ attachment styles were measured with the Relationship Scales Questionnaire, developed by Griffin and Bartholomew from the four-category model of adult attachment (Bartholomew and Horowitz, 1991; Griffin and Bartholomew, 1994) and adapted into Turkish by Sümer and Güngör (1999). The instrument contains 30 items answered on a 7-point Likert scale (1 = *not at all like me*; 7 = *very much like me*). The 17-item index comprises the four styles: secure (5 items), dismissing (5 items), preoccupied (4 items), and fearful (4 items); one item (“I am comfortable without close emotional relationships”) is reverse-coded and contributes to both the preoccupied and dismissing scores. Each subscale is scored as the arithmetic mean of its items, yielding four continuous scores ranging from 1 to 7, and each participant is assigned to the category in which she scores highest (Sümer and Güngör, 1999). Internal consistency in the Turkish adaptation study ranged from α = 0.41 to α = 0.71; in the present sample, it ranged from α = 0.59 to α = 0.82. The low end of this range is below conventional thresholds and is a known property of the instrument (Kurdek, 2002; Ravitz et al., 2010); it is addressed in the Limitations, and both the continuous and the categorical treatments are reported in the Results.

#### 2.4.3 Kerns security scale

Children’s attachment security was measured using the Security Scale developed by Kerns et al. (1996) for middle childhood and adolescence, adapted into Turkish by Sümer and Anafarta Şendağ (2009). The instrument contains 15 items in a 4-point forced-choice format, administered separately with reference to the mother and father figures; the mother-referenced form was used here, with the foster mother as the referent. Seven items (1, 3, 4, 9, 10, 13, 15) are reverse-coded, and higher scores indicate greater security. Meta-analytic evidence supports the scale’s construct validity in this age range (Brumariu et al., 2018). Internal consistency was α = 0.84 in the original development study and α = 0.84 (mother) and α = 0.88 (father) in the Turkish adaptation; in the present sample, α = 0.85 for the foster-mother form.

#### 2.4.4 Semi-structured interview schedule

Two semi-structured schedules, for foster mothers and for children, were reviewed by nine academic experts (four psychologists and one counselling specialist with expertise in attachment, one child development specialist, and three social work academics working on foster care), piloted with foster mothers and children not included in the main study, and amended accordingly. In the child interviews, the researcher first asked each child how they addressed their foster mother and used the child’s own term throughout. Both schedules are provided as Supplementary Material.

### 2.5 Ethics

Ethical approval was granted by the Non-Interventional Clinical Research Ethics Committee of the Faculty of Health Sciences, Selçuk University (decision no. 2023/467, 26 April 2023), and research permission by the Ministry of Family and Social Services (ministerial approval no. 73595336-605.01(605.01)-241, 1 June 2023). All procedures followed the Declaration of Helsinki.

Three separate consent documents were used. Foster mothers received a written information sheet and signed an informed consent form before completing any instrument. Caseworkers signed an equivalent form before completing the personal information form for the child in their charge. Children received an age-appropriate assent form in plain language, which stated that participation was voluntary, that no one would be upset if they declined, and that their name would not be shared; it was signed by the child and countersigned by the legal guardian. Participation was voluntary and unpaid at every stage, participants were told they could withdraw at any point without giving a reason, and a contact address was provided for questions.

Data were pseudonymised at entry: instruments were linked by an anonymous code rather than by name, foster mothers are identified as FM1–FM10 and children as C1–C10, and identifying details have been removed from all quotations. Because the participants are children under state protection, no individual-level data are reported.

### 2.6 Procedure

Quantitative data were collected between June and October 2023. The first author visited each province and met provincial directors and the caseworkers, through whom instruments were distributed to volunteer foster mothers and children. The caseworker completed the personal information form, the foster mother the Relationship Scales Questionnaire, and the child the Security Scale; the three were linked by an anonymous code. In total, 337, 353, and 354 of the three instruments were returned; after code matching, 314 complete dyads were retained (İstanbul 79, İzmir 45, Kahramanmaraş 38, Ankara 36, Konya 36, Gaziantep 35, Samsun 29, Malatya 16).

Qualitative data were collected afterwards. All 20 interviews, with the 10 foster mothers and the 10 children, were conducted by the first author, a male social work academic. Caseworkers arranged access to the families but were not present at any interview and took no part in the qualitative data collection. Seven dyads were interviewed face to face and three online; interviews lasted approximately 40 min with foster mothers and 25 with children. Foster mother and child were interviewed separately and in different locations; no child was interviewed in the presence of their foster mother. Face-to-face interviews took place in the foster care units because the foster mothers declined to be interviewed at home. The first author introduced himself, explained the purpose of the study, and assured participants that the information would not be used outside the research.

One field condition requires explicit disclosure. Under the undertaking required by the Ministry, audio and video recording was not permitted. The first author took contemporaneous written notes and, at the end of each interview, re-asked and clarified any passage that was incomplete or unclear before the participant left. The quotations reported below are therefore near-verbatim contemporaneous records rather than transcriptions; the implications are considered in the Limitations.

### 2.7 Data analysis

#### 2.7.1 Quantitative analysis

Analyses were conducted in SPSS 25.0. Normality was assessed through skewness and kurtosis; all scales and subscales fell within ±1.5 (Tabachnick and Fidell, 2013; Supplementary Table S2), so parametric tests were used. Group differences were tested with independent-samples t tests and one-way analysis of variance. Because group sizes were markedly unequal, the Scheffé test was used for *post hoc* comparisons, as the most conservative standard procedure and one that does not assume equal cell sizes (Scheffé, 1959). All tests were two-tailed with α = 0.05. For every inferential test, exact *p* values are reported with η^2^ (90% confidence interval from the non-central F distribution) and ω^2^ for analyses of variance, and Cohen’s d with a 95% confidence interval for t tests and *post hoc* contrasts; these were computed in Python 3 using SciPy 1.17. To control for multiple testing, tests were grouped into two families: the three confirmatory tests corresponding to the pre-specified research questions RQ1–RQ3, and the ten exploratory comparisons of child and carer characteristics. Within each family, *p* values were adjusted with the Benjamini–Hochberg false discovery rate procedure; for the confirmatory family, the more conservative Bonferroni criterion (α = 0.05/3 = 0.017) is also reported.

#### 2.7.2 Qualitative analysis

Interview notes were analysed in MAXQDA 2024 using thematic analysis (Braun and Clarke, 2006, 2021; Patton, 2014), following a sequence of data reduction, data display, and drawing and verifying conclusions (Berg and Lune, 2019). Codes were generated first, grouped into categories, and themes and subthemes defined from those categories. As a worked example, *being able to love without a blood tie* was a code within the category *feelings and thoughts about foster motherhood*, within the theme *the process before becoming a foster mother*.

Coding was carried out by the first author. This is a limitation, and the compensating procedures are stated here rather than left implicit. The coding framework and a sample of coded material were independently reviewed by the second author, a professor of social work, and by an academic specialising in child protection who had no other involvement in the study. Reviewers examined the fit between the coded extracts and the category definitions; discrepancies in category boundaries were resolved by discussion until agreement was reached, and the framework was revised twice as a result. An audit trail of codes, categories and analytic memos was maintained in MAXQDA and is available from the corresponding author. Participants’ words are presented as extended direct quotations attributed to participant identifiers so that readers can evaluate the fit between data and interpretation. No formal intercoder reliability coefficient was calculated, and none is reported; readers should weigh the qualitative findings accordingly.

Trustworthiness was addressed against the four criteria set out by Lincoln and Guba (1985) and operationalised by Shenton (2004). *Credibility* was supported by piloting, end-of-interview clarification, and the independent review of the coding framework described above. *Transferability* is supported by the description of the setting, the service model and the participants in sections 2.2 and 2.3 and in Table 2. *Dependability* rests on the audit trail and on reporting the coding sequence in enough detail to be repeated. *Confirmability* rests on grounding every interpretive claim in quoted material and on the reflexivity statement below.

##### 2.7.2.1 Reflexivity

The first author is a male social work academic who worked for seven years as a social work specialist in the Ministry’s General Directorate of Child Services. This gave access and institutional literacy, and carried a risk that participants would read him as an agent of the agency that monitors their placement, and would present their experience favourably. He therefore introduced himself as a university researcher with no current Ministry role and no influence over placement decisions, framed interviews around experience rather than evaluation of the service, and attended in analysis specifically to accounts of difficulty, conflict and regret, which are reported alongside the positive accounts.

### 2.8 Integration

The strands were integrated at three levels (Fetters et al., 2013; Bazeley, 2024): at *design*, the qualitative sample was built from the quantitative results; at *methods*, each interviewed foster mother’s attachment classification was carried into the qualitative analysis as an attribute; and at *interpretation*, findings were assembled into a joint display (Table 3) setting each quantitative result against the qualitative evidence that confirms, disconfirms or expands it, around which the Discussion is organised.

**Table 3:** Joint display: integration of the quantitative and qualitative findings.

Quantitative finding	Qualitative evidence	Relation	Interpretation
The foster mother’s attachment classification is associated with the child’s security (η^2^ = 0.113), the largest effect in the study.	Securely classified foster mothers describe attunement at first contact (FM5), dense specific accounts of shared daily time (FM4, FM10), no differentiation between foster and biological children (FM5), and the child as a source of meaning (FM5, FM10). Fearful and preoccupied foster mothers describe fear of loss and inadequacy (FM7, FM3), interaction accounts from which they are absent (FM7, FM9), and fostering as more demanding than biological parenting (FM3)	Confirms, expands	The qualitative strand supplies a behavioral mechanism, consistent with the secure base model, rather than restating the association.
Children of insecurely classified foster mothers score lower	The three children describing flat or avoidant relationships (C1, C3, C9) are the children of foster mothers classified as fearful and preoccupied; their foster mothers independently describe struggling to manage the child’s demands	Confirms (dyad level)	The correspondence within dyads argues against a shared positive response set as the sole explanation.
Earlier entry into protection is associated with higher security; small effect, η^2^ = 0.027	C2 (placed at 3) reports no memory of arrival and a sense of always having belonged; C10 (placed at 4) reports being left by force; C9 (placed at 8) reports managing distress alone.	Confirms, expands	What differs is the child’s narrative of separation rather than current caregiving, which fits a small but persistent effect.
Contact frequency is associated with security, but non-monotonically; η^2^ = 0.032	No child mentions contact frequency. Children speak instead about the emotional content of what they know of their birth family: a father in prison (C1), a mother who died (C4, C10), a family who left them by force (C10). FM7’s child flinches at knives; FM9’s child cries for her father and attempts to run away	Disconfirms; reframes	Frequency is a poor proxy for what matters to children; contact quality and emotional safety are the operative constructs.
Children’s mean security is 3.28/4 with no comparison group	Children’s metaphors for the foster mother name the haven, secure base, protection, and chosen-bond functions of an attachment figure	Expands	The children describe a functioning attachment relationship in their own terms; the absolute level remains unbenchmarked
The type of foster care service is unrelated to security (*p* = 0.398)	Kinship carers describe a distinct relational logic (“I am his grandmother, so we have a proper grandmother–grandchild relationship,” FM1); the child in that dyad reports the relationship diminishing as she ages (C1)	Diverges	The null quantitative result may mask qualitatively different relational forms, a target for future work.

## 3 Results

### 3.1 Descriptive statistics for the study variables

The mean Security Scale score of the children was M = 3.28 (SD = 0.54) on a 1–4 scale. On the Relationship Scales Questionnaire, foster mothers’ mean subscale scores were 4.38 (SD = 1.09) for secure, 4.05 (SD = 1.04) for dismissing, 4.03 (SD = 1.02) for fearful, and 3.96 (SD = 0.82) for preoccupied attachment (Table 4). Categorical assignment placed 130 foster mothers (41.4%) in the secure category, 85 (27.1%) in the dismissing category, 52 (16.6%) in the fearful category, and 47 (15.0%) in the preoccupied category (Table 5).

**Table 4:** Descriptive statistics for the study variables (*N* = 314).

Informant	Variable	Range	Min	Max	M	SD	α
Child	Kerns Security Scale	1–4	1.00	4.00	3.28	0.54	0.85
Foster mother	RSQ – Secure	1–7	1.40	7.00	4.38	1.09	0.59–0.82 ^a^
	RSQ – Fearful	1–7	1.75	6.75	4.03	1.02	
	RSQ – Preoccupied	1–7	1.25	6.75	3.96	0.82	
	RSQ – Dismissing	1–7	1.00	6.80	4.05	1.04	

RSQ, Relationship Scales Questionnaire. A range of Cronbach’s α across the four RSQ subscales in the present sample.

**Table 5:** Foster mothers’ attachment classifications (*N* = 314).

Classification	*n*	%
Secure	130	41.4
Dismissing	85	27.1
Fearful	52	16.6
Preoccupied	47	15.0

Participants were assigned to the Relationship Scales Questionnaire subscale on which they scored highest.

No normative comparison group was available, so the descriptive mean is reported without a claim about its level relative to children living with birth families.

### 3.2 RQ1: Age at entry into protection

Children’s attachment security differed significantly by the age at which they entered protection, *F*(2, 311) = 4.34, *p* = 0.014, η^2^ = 0.027, 90% CI [0.003, 0.060], ω^2^ = 0.021. This effect survives correction for multiple testing (Benjamini–Hochberg q = 0.021; Bonferroni criterion met). Scheffé *post hoc* comparisons showed that children who entered protection at age three or younger (M = 3.39, SD = 0.49) scored higher than those who entered between four and six (M = 3.22, SD = 0.53; d = 0.33, 95% CI [0.08, 0.58]) and those who entered at seven or older (M = 3.19, SD = 0.63; d = 0.37, 95% CI [0.07, 0.67]). The latter two groups did not differ from each other (d = 0.05, 95% CI [−0.25, 0.35]). The effect is small by conventional benchmarks, and the lower bound of the confidence interval is close to zero (Table 6).

**Table 6:** Children’s attachment security by age at entry into protection (RQ1).

Group	n	M	SD	*F*(2, 311)	*p*	η^2^ [90% CI]	ω^2^	*Post hoc* (Scheffé)
a. 3 years or younger	126	3.39	0.49	4.34	0.014	0.027 [0.003, 0.060]	0.021	a > b (d = 0.33 [0.08, 0.58]); a > c (d = 0.37 [0.07, 0.67])
b. 4–6 years	123	3.22	0.53					
c. 7 years or older	65	3.19	0.63					

d values are Cohen’s d with 95% confidence intervals. b and c did not differ (d = 0.05 [−0.25, 0.35]).

### 3.3 RQ2: Frequency of contact with the birth family

Attachment security differed significantly by contact frequency, *F*(4, 309) = 2.56, *p* = 0.039, η^2^ = 0.032, 90% CI [0.001, 0.060], ω^2^ = 0.020. This effect survives false discovery rate correction (q = 0.039) but not the Bonferroni criterion, and should be interpreted with corresponding caution. Scheffé comparisons located the difference between children with contact every three months (M = 3.11, SD = 0.54) and two other groups: children with no contact (M = 3.35, SD = 0.56; d = 0.43, 95% CI [0.12, 0.74]) and children with monthly contact (M = 3.34, SD = 0.51; d = 0.44, 95% CI [0.02, 0.85]).

The pattern is not monotonic. Children with no contact and with monthly contact scored almost identically (d = 0.02, 95% CI [−0.33, 0.37]), children with annual contact scored at a similar level (M = 3.33, SD = 0.53), and the two intermediate categories, every three months and every six months (M = 3.17, SD = 0.47), scored lowest. Security, therefore, does not change with contact frequency in any consistent direction. The effect is small, its confidence interval nearly includes zero, and the unequal cell sizes (*n* = 31 to 156) counsel caution (Table 7; Section 4.4).

**Table 7:** Children’s attachment security by frequency of contact with the birth family (RQ2).

Group	*n*	M	SD	*F*(4, 309)	*p*	η^2^ [90% CI]	ω^2^	*Post hoc* (Scheffé)
a. No contact	156	3.35	0.56	2.56	0.039	0.032 [0.001, 0.060]	0.020	a > c (d = 0.43 [0.12, 0.74]); b > c (d = 0.44 [0.02, 0.85])
b. Monthly	39	3.34	0.51					
c. Every three months	55	3.11	0.54					
d. Every six months	31	3.17	0.47					
e. Once a year	33	3.33	0.53					

The pattern across groups is non-monotonic: a, b, and e do not differ from one another (a vs. b, d = 0.02 [−0.33, 0.37]), while c and d are lower (see Section 4.4).

### 3.4 RQ3: Foster mother’s attachment style

Attachment security differed significantly by the foster mother’s attachment style, *F*(3, 310) = 13.16, *p* < 0.001, η^2^ = 0.113, 90% CI [0.058, 0.164], ω^2^ = 0.104. This effect survives both corrections (q < 0.001). Scheffé comparisons showed that children of foster mothers classified as secure (M = 3.50, SD = 0.42) scored higher than children of foster mothers classified as fearful (M = 3.07, SD = 0.71; d = 0.83, 95% CI [0.50, 1.16]), preoccupied (M = 3.15, SD = 0.56; d = 0.76, 95% CI [0.42, 1.10]) and dismissing (M = 3.16, SD = 0.46; d = 0.78, 95% CI [0.50, 1.06]). The three insecure categories did not differ from one another (Table 8).

**Table 8:** Children’s attachment security by foster mothers’ attachment classification (RQ3).

Group	*n*	M	SD	*F*(3, 310)	*p*	η^2^ [90% CI]	ω^2^	Post hoc (Scheffé)
a. Secure	130	3.50	0.42	13.16	< 0.001	0.113 [0.058, 0.164]	0.104	a > b (d = 0.83 [0.50, 1.16]); a > c (d = 0.76 [0.42, 1.10]); a > d (d = 0.78 [0.50, 1.06])
b. Fearful	52	3.07	0.71					
c. Preoccupied	47	3.15	0.56					
d. Dismissing	85	3.16	0.46					

Groups b, c, and d did not differ from one another.

This is by a substantial margin the largest association in the study: roughly four times the variance associated with age at entry into protection and three and a half times that associated with contact frequency, and the only one of the three effects whose confidence interval is clearly separated from zero.

Because categorical assignment based on the highest subscale mean discards information, the association was re-estimated with the four continuous subscale scores entered simultaneously in a multiple regression (Table 9; Supplementary Table S4). The four subscales jointly accounted for 14.4% of the variance in children’s attachment security, R^2^ = 0.144, adjusted R^2^ = 0.133, *F*(4, 309) = 12.97, *p* < 0.001, a larger share than the categorical analysis recovered. Only two subscales contributed independently: secure attachment, β = 0.271, t = 4.87, *p* < 0.001, sr^2^ = 0.066, and preoccupied attachment, β = −0.150, t = −2.78, *p* = 0.006, sr^2^ = 0.021. Fearful (β = −0.094, *p* = 0.083) and dismissing attachment (β = −0.051, *p* = 0.358) did not contribute once the others were held constant. Variance inflation factors ranged from 1.05 to 1.11, so the estimates are not affected by collinearity among the subscales.

**Table 9:** Multiple regression of children’s attachment security on foster mothers’ continuous attachment subscale scores (*N* = 314).

Predictor	r	β	SE	t	*p*	sr^2^	VIF
RSQ – Secure	0.325	0.271	0.056	4.87	< 0.001	0.066	1.11
RSQ – Fearful	−0.166	−0.094	0.054	−1.74	0.083	0.008	1.05
RSQ – Preoccupied	−0.216	−0.150	0.054	−2.78	0.006	0.021	1.05
RSQ – Dismissing	−0.155	−0.051	0.055	−0.92	0.358	0.002	1.09

R^2^ = 0.144, adjusted R^2^ = 0.133, *F*(4, 309) = 12.97, *p* < 0.001. RSQ, Relationship Scales Questionnaire; β, standardised regression coefficient; sr^2^, squared semi-partial correlation; VIF, variance inflation factor.

The two placement variables associated with the outcome could not be entered into the same model, and the strength of confounding they could exert can therefore only be bounded. Age at entry into protection and contact frequency are each associated with children’s security at η = 0.16 and η = 0.18, respectively. Partialling a covariate of that strength out of the observed association between foster mothers’ secure attachment and children’s security (r = 0.325) leaves a partial correlation of 0.28 to 0.30 even when the covariate is assumed to correlate as highly as 0.50 with foster mothers’ secure attachment, and no covariate correlation below 0.99 would reduce the association below r = 0.10. The association is therefore not of a magnitude that these two placement variables could account for, although a directly estimated joint model remains preferable and is identified as the first priority for future analysis.

Because internal consistency of the Relationship Scales Questionnaire subscales ranged from α = 0.59 to α = 0.82, the associations were also corrected for measurement error in both instruments. Since the lowest value could apply to any subscale, corrected estimates are reported across the full range. The correlation between foster mothers’ secure attachment and children’s security rose from r = 0.325 to between r = 0.389 (α = 0.82) and r = 0.459 (α = 0.59); the corresponding corrected correlations were −0.199 to −0.234 for fearful, −0.259 to −0.305 for preoccupied, and −0.186 to −0.219 for dismissing attachment. Unreliability in the caregiver measure therefore attenuates rather than inflates the association, and the observed estimates, including the categorical analysis, are conservative.

### 3.5 Analyses that did not reach significance

To avoid selective reporting, all analyses conducted are disclosed (Supplementary Table S3). Ten further comparisons were tested. Children’s security did not differ by sex, reason for entry into protection, duration in placement, number of biological siblings, type of foster care service, foster mother’s education, employment, or age (all *p* ≥ 0.064, all η^2^ ≤ 0.015, all |d| ≤ 0.21). Two exploratory comparisons involving siblings were significant. Children with no biological siblings scored higher than children with siblings, t(312) = 2.46, *p* = 0.015, d = 0.30, 95% CI [0.55, 0.05]; and children whose siblings were themselves in the child protection system scored lower than children whose siblings were not, t(312) = −2.02, *p* = 0.044, d = −0.24, 95% CI [−0.47, −0.02]. Both were unplanned, and neither survives correction for multiple testing within the exploratory family (Benjamini–Hochberg q = 0.146 and q = 0.214 respectively); they are reported as hypotheses rather than findings. Foster mothers aged 26–49 scored higher on secure and lower on fearful and preoccupied attachment than those aged 50 and over (d = 0.31, −0.32 and −0.40 respectively, all *p* ≤ 0.007), with no difference on dismissing attachment or by education. Because six of the eight candidate confounders showed no association with the outcome, they cannot substantially account for the RQ3 result; the two that did, age at entry into protection and contact frequency, were not modelled jointly with the foster mother’s attachment style; their possible influence is bounded in Section 3.4.

### 3.6 Qualitative findings: foster mothers

Four themes and eleven subthemes were identified (Table 10). Quotations are attributed to FM1–FM10 with each participant’s quantitative attachment classification (Table 2); two excerpts are given per subtheme and fuller quotations are in Supplementary File S4.

**Table 10:** Theme structure for the foster mother interviews.

Theme	Subtheme	Principal categories
1. The process before becoming a foster mother	1.1 Feelings and thoughts about foster motherhood	Loving without a blood tie; taking the hand of a child in need; entering an unknown process
	1.2 Motivation for becoming a foster mother	Intrinsic: maternal feeling, longing for a child, spiritual conviction, personal fulfillment. Extrinsic: social environment, encouragement by family and neighbors, contact with social services, financial expectation
	1.3 The first encounter with the child	Emotionally intense and physically detailed accounts; ordinary or administrative accounts
2. The foster motherhood process	2.1 Building the relationship with the child	Rapid adjustment and reciprocal warmth; fear, inadequacy, and crisis
	2.2 Situations of interaction with the child	Shared daily routine; shared activity; contact at moments of difficulty; absence of the carer from the account
	2.3 Foster and biological motherhood from the foster mother’s perspective	Similarities: protection, guidance, love. Differences: attachment from the heart; feelings not tasted; greater effort required
	2.4 The child’s feelings as the foster mother perceives them	Emotional closeness; belonging; feelings that impede attachment (fear of loss, separation anxiety)
3. Meanings attributed to the concept of the child	3.1 The child has a deep emotional bond	Love, innocence, sweetness, compassion
	3.2 The child as the meaning of life	Renewal, a second beginning, the joy of the house
4. The process after becoming a foster mother	4.1 Reorganisation of environmental subsystems	Extended family, neighborhood, school, health services, social services
	4.2 Making room for the child within the family	Acceptance by family members; changes in spousal and sibling roles; inclusion in family traditions

#### 3.6.1 Theme 1: The process before becoming a foster mother

##### 3.6.1.1 Feelings and thoughts about foster motherhood

Loving without a blood tie, taking the hand of a child in need, and entering an unknown process were the recurring ideas.

*Motherhood is not just about giving birth. Motherhood is not a title; it is the feeling of being able to carry the responsibility for a child's life. While we set out to touch their lives, they touched ours. (FM5, secure)*

*Being a foster family made me more sensitive and more timid. By the nature of the service model it contains fear as well, because the worry that the child could leave at any moment is dominant. (FM7, fearful)*

##### 3.6.1.2 Motivation for becoming a foster mother

Intrinsic sources (maternal feeling, longing for a child, spiritual conviction) and extrinsic ones (social environment, encouragement, contact with the social services, financial expectation) were both described.

*Because we did not have a child, I always longed for one. I wanted to take in a child, give them love, raise them with affection, and for them to know me as their mother. I wanted to give them a beautiful future. (FM7, fearful)*

*It began with my neighbour taking in a child. It saddened me that children stayed within four walls in an institution… Everyone around me also gave me courage that I could do it. (FM9, preoccupied)*

##### 3.6.1.3 The first encounter with the child

Emotional intensity at the first meeting tracked the foster mother’s classification.

*In the first meeting I made eye contact rather than physical contact… I asked permission and hugged her. When I hugged her she put her head on my chest. That was the day I felt I had become a mother. (FM5, secure)*

*We first met at the foster care unit. The staff introduced us. Since it was an institutional setting and crowded, we could not sit for very long. I cannot remember any more than that. (FM3, preoccupied)*

#### 3.6.2 Theme 2: The foster motherhood process

##### 3.6.2.1 Building the relationship with the child

Accounts divided sharply, and the division corresponded to the quantitative classification.

*We had no difficulty at all in the adjustment period. I sat and played with the children, we went shopping together, and in this way they adjusted quickly. I felt as though they had always been living with me. (FM10, secure)*

*My fear was, can I love him as much as his biological mother, can I close that gap. He was a traumatised child. Whenever he saw a knife in my hand while I was cooking he would flinch. He resisted staying in a home environment. (FM7, fearful)*

##### 3.6.2.2 Situations of interaction with the child

The texture of daily interaction also differed by classification.

*Every morning while I prepare breakfast we spend time together in the kitchen. After school I am beside him while he does his homework. In the evenings we read a book together or watch a film he likes. (FM4, secure)*

*During the week they go to school, come back, do their homework. Sometimes he talks with me and sometimes he does not. He sees his friends more often. (FM9, preoccupied)*

##### 3.6.2.3 Foster and biological motherhood from the foster mother’s perspective

Accounts of greater effort came only from foster mothers classified as preoccupied or dismissing.

*You look after your biological children anyway. But here, in a situation where you are not obliged, you love from the heart, you become attached afterwards. (FM4, secure)*

*Compared with my own biological motherhood, my experiences with my foster child have been more demanding. Complaints come right up to the door. We change classes, we change schools. (FM3, preoccupied)*

##### 3.6.2.4 The child’s feelings in the relationship, as the foster mother perceives them

Emotional closeness and feelings that impede attachment were both described.

*They have a heartfelt way of calling out, "My dear mother!" They look at me without taking their eyes off me. I ask, do you need something, and they say no, I just love you very much. (FM9, preoccupied)*

*My daughter has an intense fear of losing me. As soon as she witnesses a separation scene on television her eyes fill. She seems not to have got over her separation anxiety. (FM8, secure)*

#### 3.6.3 Theme 3: Meanings attributed to the concept of the child

##### 3.6.3.1 The child as a deep emotional bond

Affective meanings appeared across all classifications.

*For me the word child means the love of a son or daughter. It means sweetness, warmth, goodness, love, compassion. (FM9, preoccupied)*

*The word child expresses innocence and purity to me. (FM3, preoccupied)*

##### 3.6.3.2 The child as the meaning of life

Meanings of renewal were used almost only by foster mothers classified as secure.

*When the children were not here the walls always closed in on me. The hours stopped, time did not pass. Now I do not know how time passes. (FM10, secure)*

*The word child, I think, is the code of life. A person can learn a great deal from a child. (FM5, secure)*

#### 3.6.4 Theme 4: The process after becoming a foster mother

##### 3.6.4.1 Reorganisation of environmental subsystems

Extended family, neighbourhood, school and service systems were described as changing.

*Our kinship relations are intertwined. They all welcomed it very warmly. My older sister gave a large dinner for the children. After the children came, happiness and liveliness increased in our family system. (FM10, secure)*

*My husband changed a great deal. Before, he did not want me to go out, to go to my mother's. After we took the children, he himself says go, go out, so that I would not feel oppressed. (FM9, preoccupied)*

##### 3.6.4.2 Making room for the child within the family

Acceptance by family members and changes in spousal and sibling roles were described.

*When we had our biological child we called him our second child, because our first child was our foster child. The siblings were also very respectful to each other. (FM5, secure)*

*We became a big family. Sitting down at a crowded table, going on outings as a crowd, turned out to be a very beautiful feeling. (FM8, secure)*

### 3.7 Qualitative findings: children

Three themes and eleven subthemes were identified (Table 11). Quotations are attributed to C1–C10; two excerpts are given per subtheme where two children spoke to it.

**Table 11:** Theme structure for the child interviews.

Theme	Subtheme	Principal categories
1. The child’s relationship with the foster mother	1.1 Strong bonds and belonging	Feeling like part of the real family, not like an outsider, using biological language to describe the bond.
	1.2 Functionality of the relationship	Emotional support and security; communication and interaction; social support; guidance and boundaries; adaptability and flexibility
	1.3 Non-dynamic relationships	Diminishing care; oscillating closeness; self-reliance in place of help-seeking
2. The process before placement with the foster mother	2.1 A sense of family	Continuity rather than transition; welcome and preparation
	2.2 Uncertainty and anxiety	Not knowing where one is going; fear at the first meeting; not knowing one had been fostered
	2.3 Feeling safe	Prior acquaintance with the foster mother; relief at leaving the institution
	2.4 Abandonment and loneliness	Being left by one’s own family; being placed against one’s wishes
3. The perceived metaphor of foster motherhood	3.1 Safe harbour	Someone to pour out one’s heart to; sacrifice; trust; problem-solving
	3.2 Guide and light	Teacher: illumination of a dark path; help with what is not known
	3.3 Protective shield	Angel, wings, safety in her presence
	3.4 Loving from the heart	Born from the heart rather than the belly, a chosen bond

#### 3.7.1 Theme 1: The child’s relationship with the foster mother

##### 3.7.1.1 Strong bonds and belonging

*My foster mother and father are like my real family; they never made me feel like a stranger. I do not see my mother as a foster mother. My communication with my mother especially is very good. (C2)*

*Actually I feel as though my mother gave birth to me. When I close my eyes and think of the word mother, it is her I see. (C5)*

##### 3.7.1.2 Functionality of the relationship

Every child made at least one statement here

*I never suffered the lack of a mother while I was beside her. When I am upset she supports me. When I do something wrong she tries to support me. (C1)*

*She listens to me constantly. When I have any trouble I can share it with my mother without hesitation; in fact my mother mostly understands me from my facial expression. (C2)*

##### 3.7.1.3 Non-dynamic relationships

In each case the corresponding foster mother independently described difficulty.

*With my grandmother, our relationship is neither very attached nor very separate. Recently I can say there has been a decrease in the things she does for me. (C1)*

*With my mother we are sometimes good and sometimes bad. I am watching television and she says do not watch… when I say why should I not, our relationship breaks down. (C3)*

#### 3.7.2 Theme 2: The process before placement with the foster mother

##### 3.7.2.1 A sense of family

*Because I was three I do not really remember, but I felt as though I had always been together with them. When I first saw my foster mother and my unease went away, my insides fluttered, I was so very glad. (C2)*

*A welcome surprise was made at home. Before I came, food had been cooked, my room had been prepared, clothes had been bought. (C8)*

##### 3.7.2.2 Uncertainty and anxiety

*I hoped a good family would come out of it. But I also thought: will a good family or a bad family come out of it. When I first saw my foster mother, at first I was afraid. (C7)*

*I was a little worried about where we were going. When we came home I looked at my mother, I looked at my father, I was a bit shy, I could not speak very much. (C10)*

##### 3.7.2.3 Feeling safe

One child, in a kinship placement, spoke to this subtheme.

*We already knew my mother from before. When I learned I would go to her I was happier. I was going to a home for the first time; I was very happy, I was very excited. (C5)*

##### 3.7.2.4 Abandonment and loneliness

One child described the placement as a loss; this is the clearest counter-case in the data.

*At first I was very upset because my own family left me and went. I did not want to come here at all, but they left me here by force. And I tried to get myself used to living in this family. (C10)*

#### 3.7.3 Theme 3: The perceived metaphor of foster motherhood

##### 3.7.3.1 Safe harbour

*A mother, for me, is the person you share your troubles with, the person you pour out your heart to. It means sacrifice, being solution-focused, trust. (C2)*

*When I think of my foster mother, love and a great happiness come to mind. I compare my foster mother to family, and family is a beautiful thing. (C10)*

##### 3.7.3.2 Guide and light

One child spoke to this subtheme.

*My mother is more like a nursery teacher to me. She helps me with things I do not know; she is both my mother and my teacher. She is like a light that illuminates me. (C4)*

##### 3.7.3.3 Protective shield

*I compare my mother to an angel, because she is like an angel. She holds my hands, she hugs me, she really loves me. (C3)*

*My foster mother always seems to me like a guardian angel. When she is beside me, I feel safe. Her love and support make me feel as though I am spreading wings. (C4)*

##### 3.7.3.4 Loving from the heart

One child spoke to this subtheme; the image echoes the foster mothers’ language in theme 2.

*At seven, I questioned this a great deal: is a child born from the heart or from the belly? My mother said from the heart. I did not understand it then. Now I understand. (C3)*

### 3.8 Integration of the quantitative and qualitative findings

Table 3 presents a joint display setting each quantitative finding against the qualitative evidence that confirms, disconfirms, or expands it. Three patterns emerge. The association between foster mother attachment style and child security is *confirmed and explained*: securely classified foster mothers described the caregiving the secure base model specifies (attunement at first contact, dense accounts of shared time, no differentiation between foster and biological children, the child as a source of meaning), insecurely classified foster mothers described fear of loss and inadequacy and interaction accounts from which they were absent, and the three children who described flat or avoidant relationships (C1, C3, C9) were the children of fearful and preoccupied foster mothers whose own interviews independently described struggling. The finding on age at entry is *confirmed and expanded*: C2, placed at three, could not recall an arrival, while C10, placed at four, described being left by force; thus, the small statistical effect corresponds to a large difference in whether the child possesses a narrative of loss. The finding on contact frequency is *disconfirmed as a linear relationship and reframed*: no child mentioned frequency; instead, they described the emotional character of what they knew about their birth families, which concerns the meaning and quality of the connection rather than its schedule.

## 4 Discussion

This study asked whether the intergenerational transmission of attachment, established in biological parent–infant dyads, is observable in a caregiving relationship that shares neither genes nor infancy, and whether it can be detected in middle childhood using the child’s own report. The answer is that it is, that the association is of a magnitude comparable to what the transmission literature reports for biological dyads, and that it is substantially larger than the association with either of the two placement variables that dominate practice and policy discussion.

### 4.1 The foster mother’s attachment organization

Children whose foster mothers were classified as securely attached reported markedly higher attachment security than children whose foster mothers were classified as fearful, preoccupied, or dismissing, with standardized differences between 0.76 and 0.83 and 11.3% of the variance in children’s security associated with the classification. For comparison, Verhage et al. (2016) report a corrected r ≈ 0.31 between parental attachment representation and child attachment in biological dyads, accounting for roughly 10% of the variance. The association found here in non-biological dyads, in middle childhood, years after placement, is of a similar order. Because the caregiver measure has modest reliability, this is a lower bound: corrected for measurement error, the association between foster mothers’ secure attachment and children’s security lies between r = 0.39 and r = 0.46 (Section 3.4). It is consistent with the concordance Dozier et al. (2001) found among foster infants and with Stovall-McClough and Dozier's (2004) demonstration that foster infants’ attachment organized around the new caregiver’s state of mind within two months.

The finding constrains what the transmission phenomenon can be. In biological dyads, transmission is confounded with shared genes, prenatal environment, and continuous caregiving from birth; here, none of those applies, and the association persists. This is consistent with the interpretation that what is transmitted is not a trait but a pattern of caregiving conduct organized by the caregiver’s own representation of attachment, the mechanism the secure base model specifies (Schofield and Beek, 2005, 2009) and which the experimental evidence supports: Bernard et al. (2012) reduced disorganized attachment among maltreated children by intervening on the caregiver rather than the child.

The qualitative data give that mechanism concrete form, as summarised in Table 3, and the correspondence between the three children who described flat or avoidant relationships and their foster mothers’ independent accounts of struggling is the study’s most direct evidence that the association reflects something happening inside the relationship rather than a shared response tendency. That said, this is a cross-sectional association and must be read as one. In particular, the direction of influence cannot be established: an easier, more responsive child may elicit more sensitive caregiving and may also lead a foster mother to report a more secure attachment style, so that part of what appears here as transmission from carer to child may instead reflect the child’s influence on the carer. Only a longitudinal design with caregiver attachment measured at placement can separate the two (Section 4.10); this and the remaining alternatives are discussed in Section 4.9.

The recruitment route deserves particular attention because it bears directly on this finding. Both samples were reached through caseworkers, who approached families known to them, and well-functioning placements are therefore likely to be over-represented. The direction in which this biases the estimate is not self-evident. If selection favoured placements going well on both sides, carer security and child security would be jointly inflated and the association overstated; if it mainly truncated the lower end of either distribution, the restricted range would attenuate the association and the estimate would be conservative. Two features of the data suggest that the range was not seriously collapsed: a majority of foster mothers (58.6%) were classified as insecure, and children’s security scores spanned the full scale from 1.00 to 4.00. The qualitative sample was drawn from the same channel but deliberately included foster mothers from all four classifications and retained accounts of conflict, distress and self-reliance that a sample confined to well-functioning placements would not be expected to produce. A cross-province sensitivity analysis, testing whether the association holds in each recruitment site, could not be carried out with the data available to us and is identified as a priority for future analysis.

### 4.2 Age at entry into protection

Children who entered protection at three or younger reported higher attachment security than children who entered later, an effect that is statistically significant but small (η^2^ = 0.027), with a confidence interval that nearly reaches zero. The direction is consistent with Smyke et al. (2010), Bernier et al. (2004), Spieker et al. (2014), and Dozier et al. (2001), all of which found that earlier placement or stable early care produced better attachment outcomes.

The magnitude, however, deserves more caution than this literature usually receives in policy translation. The interview data suggest why the effect might be real but modest. What distinguishes early from late placement in the children’s accounts is not the quality of current caregiving but the presence or absence of a remembered narrative of separation. The difference lies in what the child carries, not in what the foster family provides, which is consistent with a small effect persisting for years in a stable placement, rather than the large effect expected if early placement determined outcomes.

### 4.3 An exploratory finding: siblings in the care system

Children with biological siblings reported lower attachment security than children without them, and security was also lower among children whose siblings were themselves in the child protection system. Neither comparison was planned and neither survives correction for multiple testing, so the pattern is offered as a hypothesis. It is nevertheless interpretable. A sibling who remains in care keeps the birth family psychologically present in a way that a sibling living with the birth family, or no sibling at all, does not, and sibling separation is itself a continuing loss that the placement does not resolve. This reading is consistent with evidence that sibling relationship quality buffers depressive symptoms among youth in foster care (McWey et al., 2023), and it suggests that the relevant variable is not the existence of siblings but where they are and whether the relationship is maintained. The study recorded the frequency of contact between children and their siblings but did not analyse it; that is the first test of this hypothesis, and it can be carried out within these data.

### 4.4 Contact with the birth family

This finding requires the most careful statement. Attachment security did differ by contact frequency, but the effect does not survive Bonferroni correction across the three confirmatory tests, and the pattern is not monotonic. Children with no contact, monthly contact, and annual contact scored at essentially the same level, while children with contact every three or six months scored lower. No linear relationship between contact frequency and attachment security exists in these data, and no policy recommendation about increasing or decreasing frequency can be derived from them.

Read against the contact literature, this is not an anomaly but a confirmation. Poitras et al. (2022a) concluded from a systematic review that visitation effects on child adjustment are inconsistent and that contact quality and parental sensitivity predict adjustment better than frequency; Ruiz-Romero et al. (2022) reached the same conclusion across 21 studies, adding that contact supports well-being when it is adequately supervised and emotionally safe and not otherwise; and Poitras et al. (2022b) found that more frequent contact with a less sensitive parent predicted more externalizing behaviour. The Málaga programme has made the point most concretely: foster families, social workers, and birth families all attribute post-visit disturbance to poor planning, unsuitable venues, and inadequate supervision rather than to contact as such (García-Martín et al., 2018; Fuentes et al., 2018), visit quality is measurable independently of frequency (García-Martín et al., 2024), and it is modifiable through structured support of birth parents (González-Pasarín et al., 2023; Bernedo and González-Pasarín, 2025a, 2025b).

If frequency is a poor proxy for quality, a frequency variable should not exhibit an orderly relationship with a child outcome, as these data show. The most defensible interpretation of the dip in the intermediate categories is that quarterly and half-yearly schedules in this system are disproportionately associated with placements in which contact is contested, irregular, or emotionally difficult, whereas both no contact and regular monthly contact reflect settled arrangements. This interpretation is *post hoc* and offered as a hypothesis for future work. What can be said with confidence is that contact frequency is not, on this evidence, a lever for improving attachment security. The interview data support this indirectly: no child raised frequency, and what children raised was the emotional content of what they knew of their birth families, consistent with Salas Martínez et al.'s (2016) finding that children, foster parents, and social workers evaluate the same visits very differently.

Contact must also be framed as a right, not only as a variable. Under the Convention on the Rights of the Child (United Nations, 1989), a child separated from their parents is entitled to maintain personal relations and direct contact with them regularly unless this is contrary to the child’s best interests. A measured association between contact frequency and an attachment score does not, on its own, license restricting a right. The implication of these findings is therefore about how contact is planned, prepared, and supported, not about whether it should occur.

### 4.5 What the children’s accounts add

The metaphors children used for foster motherhood, safe harbour, guide and light, protective shield, and loving from the heart, name the functions of an attachment figure with unusual precision: haven, secure base, protection, and a bond understood as chosen rather than given. These children were describing an internal working model of a caregiver who is available, who can be returned to under stress, and whose commitment is not contingent on biology, at the developmental stage when attachment reorganizes around availability and open communication rather than proximity (Kerns et al., 1996; Kerns and Brumariu, 2014).

The *loving-from-the-heart metaphor is the most theoretically interesting because children and foster mothers arrived at the same image independently: FM4 spoke of loving from the heart even when* she was not obliged to, and C3 asked whether a child is born from the heart or from the belly. This shared vocabulary appears to be how both parties resolve the absence of a biological tie into a legitimate claim of kinship, echoing Diogo and Branco's (2017) finding among Portuguese foster mothers. The negative cases are equally informative: C1, C3, C9, and C10 described diminishing care, an oscillating relationship, self-reliance in place of help-seeking, and placement experienced as abandonment. These are the qualitative counterparts of the insecure groups in Table 8, and a study drawn from all four attachment classifications that reported only warm accounts would have failed its own design.

### 4.6 The foster mothers’ route into the role

Two foster mother themes concern the periods before and after placement. The motivations described divided into intrinsic sources (maternal feeling, a longing for a child, spiritual conviction) and extrinsic ones (encouragement from family and neighbours, contact with the social services, in one case financial expectation), which maps onto the child-centred and self-focused distinction of Rhodes et al. (2006) and Cole (2005) found that reasons for fostering predict the quality of the relationship subsequently formed. Because the Turkish model pays approved families a monthly maintenance allowance, the question of whether payment functions as a motive rather than a support is a live one, and it has been raised for alternative care systems generally (Engle et al., 2011). These data cannot answer it: motivation was explored only in the interviews and was not measured in the survey, so the prevalence of financially driven fostering in this population is unknown. What can be said is that the one account in which payment figured was not otherwise distinctive, and that the qualitative material as a whole is dominated by child-centred and spiritual motives rather than material ones. The theme of entering an unknown process is where the service model enters the psychology of the relationship: the two foster mothers who described fearing the child’s removal (FM7, FM3) were both classified as insecure, and both described building the relationship as frightening. Dozier and Lindhiem (2006) identified commitment as a construct specific to non-biological caregiving, and Turner et al. (2023) found that foster carers sustain investment despite considerable difficulty; these accounts suggest that the structural impermanence of the placement, a feature of the model rather than the carer, is one condition under which commitment becomes hard to sustain, and one that is modifiable through how placements are communicated and supported. The fourth theme, the reorganization of the household and its wider systems, points towards assessment and support that address the household and its network rather than the carer alone.

### 4.7 Findings in the Turkish cultural context

Two features of the setting shape how these results should be read. First, care of children without parents is framed in Türkiye through religious obligation as much as through welfare policy, and this was audible in the interviews: spiritual conviction was among the most frequently named intrinsic motives, and foster mothers described the child as entrusted to them rather than given to them. That framing supports commitment without requiring the fiction of biological kinship, which may be why the *loving from the heart* metaphor was produced independently by foster mothers and children as a way of establishing a legitimate claim to one another. Second, the extended family is an active party to the placement rather than a background condition. Foster mothers described relatives organising welcomes, spouses changing their conduct, and kinship networks absorbing the child, and kinship or close-environment placements accounted for 17.5% of the sample. Where the caregiving unit is the household and its network rather than the dyad, an attachment measured only at the level of the foster mother may understate the resources available to the child (Akan Ellis, 2021). Both features caution against transferring these estimates directly to systems in which fostering is organised as a contractual service between the state and an individual carer.

### 4.8 Implications for policy and practice

Three implications follow, each calibrated to the magnitude of the corresponding finding.

#### 4.8.1 Attachment-informed assessment and support of foster carers

This rests on the strongest finding. Foster carer assessment in Türkiye currently examines material circumstances, household composition, motivation, and social investigation; these findings suggest that the caregiver’s own attachment organization is associated with several times as much variance in child outcomes as either placement timing or contact arrangements. That does not warrant screening out prospective carers based on an attachment measure, which would be psychometrically indefensible with instruments of this reliability and ethically dubious. It warrants using attachment as a *support* variable: identifying carers likely to need more intensive help with the relationship, and giving it to them. Attachment-based caregiver interventions, as evaluated by Bernard et al. (2012), have demonstrated effects on children’s attachment organization and are a far more specific proposal than generic parenting training.

#### 4.8.2 Support at the point of placement, especially for later-placed children

Children placed after infancy carry a narrative of separation into the placement, and their foster mothers described precisely this as the source of their own sense of inadequacy. Preparing both parties for the first encounter and supporting them through the first months is where the earlier-placement advantage might be partly compensated.

#### 4.8.3 Contact quality rather than contact frequency

These data provide no basis for changing the frequency of contact. They provide a basis for attending to how contact is planned, prepared, located, and supervised, and for supporting birth parents before and after visits, which the Spanish intervention studies show is feasible; the observational quality index of García-Martín et al. (2024) offers a practicable instrument for directorates that currently record only frequency.

### 4.9 Limitations

The design is cross-sectional, and the associations are not causal. Reverse causation (an affectionate, easy child raising a caregiver’s self-reported security) and selection (Ministry screening favouring both secure carers and the placement of less disturbed children with them) cannot be excluded, and placement stability, the strongest known predictor in this literature, was not recorded. The foster mother’s four attachment subscales are now modelled jointly (Section 3.4), but age at entry into protection and contact frequency could not be entered into the same model, so the caregiver’s incremental contribution is bounded by sensitivity analysis rather than estimated directly. A jointly specified model remains the preferable analysis and is the first item under Future research. Both instruments are self-report, and although they were completed by different informants with administrative data recorded by the caseworker, common method variance remains a residual threat.

The Relationship Scales Questionnaire showed internal consistency as low as α = 0.59 on one subscale, a documented weakness of the instrument (Kurdek, 2002; Ravitz et al., 2010); this attenuates rather than inflates the observed associations (Section 3.4), but categorical assignment by highest subscale mean also discards information and produced unequal groups. No factor structure or measurement invariance was tested, and the Security Scale captures the child’s perception of the relationship rather than attachment organization assessed observationally. Recruitment through caseworkers probably over-represents well-functioning placements (see Section 4.1), no comparison group of children living with birth families was available, and the sample is confined to eight provinces, to children aged 9–12 in placement for at least a year, and to foster mothers. The foster mother’s length of experience in the role was recorded for the interview sample but was not asked in the survey form, so it could not be examined quantitatively. Since experience plausibly shapes both caregiving behaviour and the carer’s own account of it, this is a substantive omission and a variable that any replication should collect. The design had 80% power to detect η^2^ ≥ 0.034, so the effects for RQ1 and RQ2 sit at the edge of reliable detection, cell sizes were unequal, and non-independence among children sharing a foster mother was not modelled.

In the qualitative strand, coding was performed by a single researcher without an intercoder reliability coefficient, the Ministry’s prohibition on recording means quotations are contemporaneous notes rather than transcripts, and the first author’s former Ministry employment and the directorate interview setting may have inclined participants towards favourable accounts.

### 4.10 Future research

Five lines of work follow directly. The first is a reanalysis of these data in a single multivariable model entering age at entry into protection and contact frequency alongside the foster mother’s continuous attachment scores, to estimate the caregiver’s incremental contribution, together with a cross-province sensitivity analysis testing whether the association holds in each recruitment site. Beyond that, a longitudinal design following newly placed children over two years with caregiver attachment measured at baseline would separate transmission from reverse causation; Kliewer-Neumann et al. (2023) show this is feasible and that the relevant change occurs within months. An observational measure of foster carer sensitivity alongside the self-report measures would test whether sensitivity mediates the association as it partially does in biological dyads (Verhage et al., 2016) and would break the common-method dependence. Contact should be measured by quality rather than frequency, using an instrument such as the observational checklist of García-Martín et al. (2024), which would test the interpretation offered in Section 4.4 directly. Finally, including foster fathers and the father-referenced form of the Security Scale would establish whether the association is specific to the maternal relationship or a property of the household.

## 5 Conclusion

In a national sample of 314 foster mother–child dyads in Türkiye, foster mothers’ attachment styles were associated with several times more variance in the attachment security of children aged 9–12 than either age at entry into protection or frequency of contact with the birth family, at a magnitude comparable to the meta-analytic benchmark for biological dyads. This suggests that the intergenerational transmission of attachment is not contingent on shared biology or shared infancy and remains detectable in middle childhood. Foster mothers’ and children’s accounts describe the mechanism in convergent terms: securely attached foster mothers described attuned, undifferentiated caregiving, and their children described a safe harbour, a guide, a shield, and someone who loves from the heart. The association between contact frequency and security was not linear, and no recommendation about frequency follows; what follows is that contact quality is where attention belongs. For practice, the attachment organization of the person who will do the caregiving is the most consequential variable identified here, and supporting it is a more promising target than adjusting the parameters of the placement.

## Data Availability

The datasets generated and analysed for this study are not publicly available because the participants are children in state protection and the research permission granted by the Ministry of Family and Social Services does not permit open deposition of individual-level data. De-identified data and the analysis syntax are available from the corresponding author on reasonable request, subject to approval by the Ministry. Requests to access the datasets should be directed to Cezmi Ervüz, cervuz013@gmail.com. The interview schedules and the COREQ checklist are provided as Supplementary Material.

## References

[ref1] Ainsworth M. D. S. (1979). Infant–mother attachment. Am. Psychol. 34, 932–937. doi: 10.1037/0003-066X.34.10.932, 517843

[ref2] Akan Ellis B. (2021). Norm localization and contestation: the politics of foster children in Turkey. Genealogy 5:25. doi: 10.3390/genealogy5010025

[ref3] Bartholomew K. Horowitz L. M. (1991). Attachment styles among young adults: a test of a four-category model. J. Pers. Soc. Psychol. 61, 226–244. doi: 10.1037/0022-3514.61.2.226, 1920064

[ref4] Bazeley P. (2024). Conceptualizing integration in mixed methods research. J. Mixed Methods Res. 18, 225–234. doi: 10.1177/15586898241253636

[ref5] Berg B. L. Lune H. (2019). Qualitative Research Methods for the Social Sciences. 9th Edn Harlow: Pearson.

[ref6] Bernard K. Dozier M. Bick J. Lewis-Morrarty E. Lindhiem O. Carlson E. (2012). Enhancing attachment organization among maltreated children: results of a randomized clinical trial. Child Dev. 83, 623–636. doi: 10.1111/j.1467-8624.2011.01712.x, 22239483 PMC3305815

[ref7] Bernedo I. M. González-Pasarín L. (2025a). Impact of visits on fostered children's and families' well-being: views of birth families, foster families, and social workers. J. Public Child Welfare 19, 1136–1158. doi: 10.1080/15548732.2024.2399585

[ref8] Bernedo I. M. González-Pasarín L. (2025b). Improving the quality of contacts during foster care: learnings from foster families. Br. J. Soc. Work. 55, 1617–1635. doi: 10.1093/bjsw/bcaf006

[ref9] Bernier A. Ackerman J. P. Stovall-McClough K. C. (2004). Predicting the quality of attachment relationships in foster care dyads from infants' initial behaviors upon placement. Infant Behav. Dev. 27, 366–381. doi: 10.1016/j.infbeh.2004.01.001

[ref10] Bowlby J. (1958). The nature of the child's tie to his mother. Int. J. Psychoanal. 39, 350–373. 13610508

[ref11] Braun V. Clarke V. (2006). Using thematic analysis in psychology. Qual. Res. Psychol. 3, 77–101. doi: 10.1191/1478088706qp063oa

[ref12] Braun V. Clarke V. (2021). One size fits all? What counts as quality practice in (reflexive) thematic analysis? Qual. Res. Psychol. 18, 328–352. doi: 10.1080/14780887.2020.1769238

[ref13] Brumariu L. E. Kerns K. A. (2010). Parent–child attachment and internalizing symptoms in childhood and adolescence: a review of empirical findings and future directions. Dev. Psychopathol. 22, 177–203. doi: 10.1017/S0954579409990344, 20102655

[ref14] Brumariu L. E. Madigan S. Giuseppone K. R. Abtahi M. M. Kerns K. A. (2018). The security scale as a measure of attachment: meta-analytic evidence of validity. Attach. Hum. Dev. 20, 600–625. doi: 10.1080/14616734.2018.1433217, 29402180

[ref15] Cole S. A. (2005). Foster caregiver motivation and infant attachment: how do reasons for fostering affect relationships? Child Adolesc. Soc. Work J. 22, 441–457. doi: 10.1007/s10560-005-0021-x

[ref16] Creswell J. W. (2002). Educational Research: Planning, Conducting, and Evaluating Quantitative and Qualitative Research. Upper Saddle River, NJ: Prentice Hall.

[ref17] Creswell J. W. Plano Clark V. L. (2011). Designing and Conducting Mixed Methods Research. 2nd Edn Thousand Oaks, CA: Sage.

[ref18] Dalgaard N. T. Reich J. M. Jensen J. K. Hillman S. Pontoppidan M. (2025). Exploring attachment representations and traumatic reenactment in foster children. Attach. Hum. Dev. 27, 480–506. doi: 10.1080/14616734.2025.2503707, 40355285

[ref19] Diogo E. Branco F. (2017). Being a foster family in Portugal: motivations and experiences. Societies 7:37. doi: 10.3390/soc7040037

[ref20] Dozier M. Lindhiem O. (2006). This is my child: differences among foster parents in commitment to their young children. Child Maltreat. 11, 338–345. doi: 10.1177/1077559506291263, 17043318

[ref21] Dozier M. Stovall K. C. Albus K. E. Bates B. (2001). Attachment for infants in foster care: the role of caregiver state of mind. Child Dev. 72, 1467–1477. doi: 10.1111/1467-8624.00360, 11699682

[ref22] Engle P. L. Groza V. K. Groark C. J. Greenberg A. Bunkers K. M. Muhamedrahimov R. J. (2011). VIII. The situation for children without parental care and strategies for policy change. Monogr. Soc. Res. Child Dev. 76, 190–222. doi: 10.1111/j.1540-5834.2011.00633.x

[ref23] Fetters M. D. Curry L. A. Creswell J. W. (2013). Achieving integration in mixed methods designs: principles and practices. Health Serv. Res. 48, 2134–2156. doi: 10.1111/1475-6773.12117, 24279835 PMC4097839

[ref24] Fuentes M. J. Bernedo I. M. Salas M. D. García-Martín M. A. (2018). What do foster families and social workers think about children's contact with birth parents? A focus group analysis. Int. Soc. Work. 62, 1416–1430. doi: 10.1177/0020872818775475

[ref25] García-Martín M. Á. Bernedo I. M. Salas M. D. González-Pasarín L. (2024). Indicators of contact visit quality in non-kinship foster care: an observational checklist. Child. Soc. 38, 524–546. doi: 10.1111/chso.12723

[ref26] García-Martín M. Á. Fuentes M. J. Bernedo I. M. Salas M. D. (2018). The views of birth families regarding access to visits in foster care. J. Soc. Work. 19, 173–191. doi: 10.1177/1468017318757399

[ref27] González-Pasarín L. Bernedo I. M. García-Martín M. A. (2023). A qualitative study about changes that parents experience through a pilot parenting support program to improve the quality of contact visits in non-kinship foster care. Child Youth Serv. Rev. 148:106871. doi: 10.1016/j.childyouth.2023.106871

[ref28] Griffin D. W. Bartholomew K. (1994). Models of the self and other: fundamental dimensions underlying measures of adult attachment. J. Pers. Soc. Psychol. 67, 430–445. doi: 10.1037/0022-3514.67.3.430

[ref29] Groh A. M. Fearon R. P. Bakermans-Kranenburg M. J. van IJzendoorn M. H. Steele R. D. Roisman G. I. (2014). The significance of attachment security for children's social competence with peers: a meta-analytic study. Attach. Hum. Dev. 16, 103–136. doi: 10.1080/14616734.2014.883636 PMC4021853 24547936

[ref30] Guetterman T. C. Fetters M. D. Creswell J. W. (2015). Integrating quantitative and qualitative results in health science mixed methods research through joint displays. Ann. Fam. Med. 13, 554–561. doi: 10.1370/afm.1865, 26553895 PMC4639381

[ref31] Jorjadze N. Bovenschen I. Spangler G. (2023). Attachment behavior, attachment representations, and symptoms of reactive attachment disorder and disinhibited social engagement disorder in foster children with different preplacement experiences. Dev. Child Welfare 5, 138–157. doi: 10.1177/25161032231193168

[ref32] Karasar N. (2011). Bilimsel Araştırma Yöntemi [Scientific Research Method]. Ankara: Nobel Yayınları.

[ref33] Karaveli R. Erükçü Akbaş G. (2026). An evaluation of the child protection system in Türkiye: challenges, solutions, and opportunities. Child Youth Serv. Rev. 180:108665. doi: 10.1016/j.childyouth.2025.108665

[ref34] Kelebek Ö. Kucur F. (2025). Examining children's homes in light of Türkiye's evolving child welfare system. Child Youth Serv. Rev. 171:108193. doi: 10.1016/j.childyouth.2025.108193, 42574925

[ref35] Kerns K. A. Brumariu L. E. (2014). Is insecure parent–child attachment a risk factor for the development of anxiety in childhood or adolescence? Child Dev. Perspect. 8, 12–17. doi: 10.1111/cdep.12054, 24660023 PMC3960076

[ref36] Kerns K. A. Klepac L. Cole A. (1996). Peer relationships and preadolescents' perceptions of security in the child–mother relationship. Dev. Psychol. 32, 457–466. doi: 10.1037/0012-1649.32.3.457

[ref37] Kliewer-Neumann J. D. Zimmermann J. Bovenschen I. Gabler S. Lang K. Spangler G. . (2023). Attachment disorder symptoms in foster children: development and associations with attachment security. Child Adolesc. Psychiatry Ment. Health 17:98. doi: 10.1186/s13034-023-00636-5, 37568160 PMC10422696

[ref38] Kurdek L. A. (2002). On being insecure about the assessment of attachment styles. J. Soc. Pers. Relat. 19, 811–834. doi: 10.1177/0265407502196005

[ref39] León E. Jiménez-Morago J. M. Muñoz-Silva A. (2017). Contact between birth parents and children in kinship care in a sample from Spain. Child Fam. Soc. Work 22, 1075–1083. doi: 10.1111/cfs.12327

[ref40] Lincoln Y. S. Guba E. G. (1985). Naturalistic Inquiry. Beverly Hills, CA: Sage.

[ref41] Malterud K. Siersma V. D. Guassora A. D. (2016). Sample size in qualitative interview studies: guided by information power. Qual. Health Res. 26, 1753–1760. doi: 10.1177/1049732315617444, 26613970

[ref42] Martínez-Usarralde M.-J. Conchell R. Villar M. Pérez-Tabernero L. (2025). The need for better attachment bonds between institutional caregivers and children in residential care: a systematic review. Behav. Sci. 15:245. doi: 10.3390/bs15030245, 40150140 PMC11939810

[ref43] McWey L. M. (2004). Predictors of attachment styles of children in foster care: an attachment theory model for working with families. J. Marital. Fam. Ther. 30, 439–452. doi: 10.1111/j.1752-0606.2004.tb01254.x, 15532252

[ref44] McWey L. M. Cui M. Wojciak A. S. (2023). The importance of sibling relationships in buffering against depressive symptoms of youth in foster care. Fam. Soc. 104, 465–475. doi: 10.1177/10443894221141594

[ref45] Ministry of Family and Social Services (2024). Koruyucu Aile İstatistikleri [Foster Family Statistics] Ankara Republic of Türkiye Ministry of Family and Social Services. Available online at: https://aile.gov.tr/koruyucuaile/illere-gore-koruyucu-aile-istatistikleri/ (accessed June 7, 2024).

[ref46] Patton M. Q. (2014). Nitel Araştırma ve Değerlendirme Yöntemleri [Qualitative Research and Evaluation Methods]. Ankara: Pegem Akademi.

[ref47] Poitras K. Porlier S. Tarabulsy G. M. (2022a). Child's adjustment and parent–child contact after child placement into foster care: a systematic review. J. Public Child Welfare 16, 575–606. doi: 10.1080/15548732.2021.1940416

[ref48] Poitras K. Tarabulsy G. M. Varela N. (2022b). Contact with biological parents following placement in foster care: associations with preschool child externalizing behavior. Clin. Child Psychol. Psychiatry 27, 466–479. doi: 10.1177/13591045211049295, 34632816 PMC9047105

[ref49] Ravitz P. Maunder R. Hunter J. Sthankiya B. Lancee W. (2010). Adult attachment measures: a 25-year review. J. Psychosom. Res. 69, 419–432. doi: 10.1016/j.jpsychores.2009.08.006, 20846544

[ref50] Rhodes K. Cox M. E. Orme J. G. Coakley T. (2006). Foster parents' reasons for fostering and foster family utilization. J. Sociol. Soc. Welfare 33, 105–126. doi: 10.15453/0191-5096.3206

[ref51] Ruiz-Romero K. J. Salas M. D. Fernández-Baena F. J. González-Pasarín L. (2022). Is contact with birth parents beneficial to children in non-kinship foster care? A scoping review of the evidence. Child Youth Serv. Rev. 143:106658. doi: 10.1016/j.childyouth.2022.106658

[ref52] Salas Martínez M. D. Fuentes M. J. Bernedo I. M. García-Martín M. A. (2016). Contact visits between foster children and their birth family: the views of foster children, foster parents, and social workers. Child Fam. Soc. Work 21, 473–483. doi: 10.1111/cfs.12163

[ref53] Scheffé H. (1959). The Analysis of Variance. New York, NY: John Wiley & Sons.

[ref54] Schofield G. Beek M. (2005). Providing a secure base: parenting children in long-term foster family care. Attach. Hum. Dev. 7, 3–25. doi: 10.1080/14616730500049019, 15981613

[ref55] Schofield G. Beek M. (2009). Growing up in foster care: providing a secure base through adolescence. Child Fam. Soc. Work 14, 255–266. doi: 10.1111/j.1365-2206.2008.00592.x

[ref56] Shenton A. K. (2004). Strategies for ensuring trustworthiness in qualitative research projects. Educ. Inf. 22, 63–75. doi: 10.3233/EFI-2004-22201

[ref57] Smyke A. T. Zeanah C. H. Fox N. A. Nelson C. A. Guthrie D. (2010). Placement in foster care enhances the quality of attachment among young institutionalized children. Child Dev. 81, 212–223. doi: 10.1111/j.1467-8624.2009.01390.x PMC4098033 20331663

[ref58] Spieker S. J. Oxford M. L. Fleming C. B. (2014). Permanency outcomes for toddlers in child welfare two years after a randomized trial of a parenting intervention. Child Youth Serv. Rev. 44, 201–206. doi: 10.1016/j.childyouth.2014.06.017, 25125769 PMC4128499

[ref59] Stovall-McClough K. C. Dozier M. (2004). Forming attachments in foster care: infant attachment behaviors during the first 2 months of placement. Dev. Psychopathol. 16, 253–271. doi: 10.1017/S0954579404044505, 15487595

[ref60] Sümer N. Anafarta Şendağ M. (2009). Orta çocukluk döneminde ebeveynlere bağlanma, benlik algısı ve kaygı [Attachment to parents, self-perception and anxiety in middle childhood]. Turk. Psikol. Derg. 24, 86–101.

[ref61] Sümer N. Güngör D. (1999). Yetişkin bağlanma stilleri ölçeklerinin Türk örneklemi üzerinde psikometrik değerlendirmesi ve kültürlerarası karşılaştırma [Psychometric evaluation of adult attachment style scales on a Turkish sample and a cross-cultural comparison]. Turk. Psikol. Derg. 14, 71–106.

[ref62] Tabachnick B. G. Fidell L. S. (2013). Using Multivariate Statistics. 6th Edn Boston, MA: Pearson.

[ref63] Tong A. Sainsbury P. Craig J. (2007). Consolidated criteria for reporting qualitative research (COREQ): a 32-item checklist for interviews and focus groups. Int. J. Qual. Health Care 19, 349–357. doi: 10.1093/intqhc/mzm042, 17872937

[ref64] Turner F. Kainth G. MacDonald S. O'Connor R. Crawford K. Minnis H. (2023). "I will commit to this child as much as I can for the time that they are with me": a qualitative examination of how foster carer commitment relates to short-term foster care for young children following abuse and neglect. Child Abuse Negl. 135:105983. doi: 10.1016/j.chiabu.2022.105983, 36525826

[ref65] United Nations (1989). Convention on the Rights of the Child. United Nations Treaty Series 1577, 3. New York, NY: United Nations.

[ref66] van IJzendoorn M. H. (1995). Adult attachment representations, parental responsiveness, and infant attachment: a meta-analysis on the predictive validity of the adult attachment interview. Psychol. Bull. 117, 387–403. doi: 10.1037/0033-2909.117.3.387, 7777645

[ref67] Verhage M. L. Schuengel C. Madigan S. Fearon R. M. P. Oosterman M. Cassibba R. . (2016). Narrowing the transmission gap: a synthesis of three decades of research on intergenerational transmission of attachment. Psychol. Bull. 142, 337–366. doi: 10.1037/bul0000038, 26653864

