# Supplementary material for: Foster mothers’ attachment styles and children’s attachment security in middle childhood: a mixed-methods study of the Turkish foster family service model
Source: Front Psychol. 2026 Oct 12;17:1986177. doi: 10.3389/fpsyg.2026.1986177 (PMC13696446; doi:10.3389/fpsyg.2026.1986177)
Supplement: Supplementary file 1 [file Data_Sheet_1.pdf]

Supplementary Material

Foster mothers' attachment styles and children's attachment security in middle childhood: a mixed-methods study of the Turkish foster family service model

Cezmi Ervüz and Özlem Karakuş. Frontiers in Psychology, manuscript 1986177.

Section numbers refer to the published article. Tables S1–S4 and Files S1–S4 are cited in sections 2.1, 2.3.1, 2.4.4, 2.7.1, 3.4, 3.5 and 3.6 of the article.

Supplementary Table S1. Quota sampling allocation and realised sample by province

Province	Accessible population (children aged 9–12 in the service model)	Weight (N ÷ 682)	Target quota	Realised sample (complete dyads)	% of realised sample
İstanbul	192	0.28	69	79	25.2
İzmir	107	0.16	40	45	14.3
Ankara	86	0.13	32	36	11.5
Konya	73	0.11	27	36	11.5
Samsun	65	0.09	22	29	9.2
Gaziantep	64	0.09	22	35	11.1
Kahramanmaraş	61	0.09	22	38	12.1
Malatya	34	0.05	12	16	5.1
Total	682	1.00	246	314	100.0

The accessible population (N = 682) was defined across eight provinces selected for geographic diversity and for the number of children in the foster family service model. The minimum sample size of 246 dyads was obtained from the finite-population formula $n = (N \times t^2 \times p \times q) \div (d^2(N - 1) + t^2 \times p \times q)$ with $t = 1.96$, $p = q = 0.50$ and $d = 0.05$, and was allocated across provinces in proportion to their share of the accessible population. The realised sample of 314 complete dyads exceeded the quota in every province and represents 13.2% of the 2,379 children aged 9–12 in the service model nationally.

Supplementary Table S2. Skewness and kurtosis coefficients for all scales and subscales

Scale / subscale	n	Skewness	SE	Kurtosis	SE
Kerns Security Scale (child)	314	−0.915	0.138	0.737	0.274
RSQ – Secure (foster mother)	314	−0.064	0.138	−0.305	0.274
RSQ – Fearful (foster mother)	314	0.094	0.138	−0.278	0.274
RSQ – Preoccupied (foster mother)	314	−0.003	0.138	0.822	0.274
RSQ – Dismissing (foster mother)	314	−0.554	0.138	0.678	0.274

RSQ, Relationship Scales Questionnaire. All coefficients fall within ±1.5, the criterion adopted from Tabachnick and Fidell (2013), so parametric tests were used throughout (section 2.7.1).

Supplementary Table S3. Full results of all non-significant and exploratory analyses

Panel A. Children's attachment security by child and placement characteristics

Comparison	n per group	M (SD)	Test	p	Effect size	BH q
Child's sex (boys vs girls)	134 / 180	3.33 (0.52) / 3.25 (0.56)	t(312) = 1.16	0.247	d = 0.13	—
Reason for entry into protection	6 groups (30–137)	3.22–3.43	F(5, 308) = 0.93	0.463	η² = 0.015	—
Duration in the foster placement	86 / 126 / 102	3.28 (0.56) / 3.29 (0.55) / 3.28 (0.52)	F(2, 311) = 0.01	0.989	η² < 0.001	—
Number of biological siblings (among children with siblings)	76 / 106 / 46	3.21 (0.53) / 3.27 (0.51) / 3.22 (0.59)	F(2, 225) = 0.34	0.713	η² = 0.003	—
Type of foster care service	31 / 55 / 228	3.18 (0.62) / 3.25 (0.52) / 3.31 (0.54)	F(2, 311) = 0.92	0.398	η² = 0.006	—
Foster mother's education	148 / 43 / 69 / 54	3.27 (0.51) / 3.26 (0.55) / 3.31 (0.53) / 3.31 (0.63)	F(3, 310) = 0.21	0.892	η² = 0.002	—
Foster mother's employment (not employed vs employed)	213 / 101	3.25 (0.53) / 3.36 (0.54)	t(312) = −1.86	0.064	d = −0.21	—
Foster mother's age (26–49 vs 50+)	144 / 170	3.33 (0.48) / 3.24 (0.58)	t(312) = 1.42	0.156	d = 0.17	—
Biological siblings (no vs yes)	86 / 228	3.40 (0.56) / 3.24 (0.53)	t(312) = 2.46	0.015	d = 0.30 [0.05, 0.55]	0.146
Siblings in the child protection system (yes vs no)	182 / 132	3.23 (0.52) / 3.36 (0.56)	t(312) = −2.02	0.044	d = −0.24 [−0.47, −0.02]	0.214

Panel B. Foster mothers' attachment subscale scores by carer and child characteristics

Comparison	n per group	M (SD)	Test	p	Effect size
Carer age (26–49 vs 50+): secure	144 / 170	4.56 (1.05) / 4.23 (1.11)	t(312) = 2.71	0.007	d = 0.31
Carer age: fearful	144 / 170	3.86 (0.97) / 4.18 (1.04)	t(312) = −2.77	0.006	d = −0.32
Carer age: preoccupied	144 / 170	3.79 (0.82) / 4.11 (0.80)	t(312) = −3.52	< 0.001	d = −0.40
Carer age: dismissing	144 / 170	3.97 (0.98) / 4.11 (1.08)	t(312) = −1.22	0.223	d = −0.14
Carer education: secure	148 / 43 / 69 / 54	4.41 (1.14) / 4.23 (1.24) / 4.39 (1.04) / 4.38 (0.94)	F(3, 310) = 0.31	0.820	η² = 0.003

Carer education: fearful	148 / 43 / 69 / 54	4.15 (0.97) / 4.10 (1.17) / 3.91 (0.94) / 3.80 (1.10)	F(3, 310) = 2.05	0.107	η² = 0.019
Carer education: preoccupied	148 / 43 / 69 / 54	4.03 (0.88) / 3.79 (0.76) / 3.89 (0.82) / 4.01 (0.73)	F(3, 310) = 1.22	0.303	η² = 0.012
Carer education: dismissing	148 / 43 / 69 / 54	4.07 (1.00) / 4.15 (1.12) / 4.03 (1.13) / 3.96 (1.02)	F(3, 310) = 0.28	0.841	η² = 0.003
Carer employment (not employed vs employed): secure	213 / 101	4.40 (1.16) / 4.34 (0.95)	t(312) = 0.41	0.682	d = 0.05
Carer employment: fearful	213 / 101	4.15 (1.00) / 3.79 (1.02)	t(312) = 2.95	0.003	d = 0.35
Carer employment: preoccupied	213 / 101	3.94 (0.85) / 4.01 (0.77)	t(312) = −0.64	0.525	d = −0.08
Carer employment: dismissing	213 / 101	4.03 (1.03) / 4.10 (1.07)	t(312) = −0.61	0.540	d = −0.07
Child’s sex: secure	134 / 180	4.49 (1.08) / 4.30 (1.10)	t(312) = 1.55	0.121	d = 0.17
Child’s sex: fearful	134 / 180	4.08 (1.09) / 4.00 (0.97)	t(312) = 0.72	0.473	d = 0.08
Child’s sex: preoccupied	134 / 180	3.92 (0.77) / 4.00 (0.87)	t(312) = −0.85	0.394	d = −0.10
Child’s sex: dismissing	134 / 180	4.02 (0.92) / 4.08 (1.13)	t(312) = −0.48	0.629	d = −0.05

Panel C. Correction for multiple testing

Tests were assigned to two families (section 2.7.1). The confirmatory family comprised the three tests corresponding to RQ1–RQ3: age at entry into protection, $F(2, 311) = 4.34$, $p = 0.014$, BH $q = 0.021$, Bonferroni criterion ($\alpha = 0.017$) met; contact frequency, $F(4, 309) = 2.56$, $p = 0.039$, BH $q = 0.039$, Bonferroni criterion not met; foster mother’s attachment classification, $F(3, 310) = 13.16$, $p < 0.001$, BH $q < 0.001$, Bonferroni criterion met. The exploratory family comprised the ten comparisons in Panel A; the two significant comparisons do not survive Benjamini–Hochberg correction within that family ($q = 0.146$ and $q = 0.214$). Panel B analyses concern the carer measure rather than the child outcome and are reported for completeness; they were not included in either family.

Notes. All tests are two-tailed. Cohen’s d is reported with the direction given by the order of the groups named in the first column; 95% confidence intervals are given for the two significant exploratory comparisons, which are discussed in sections 3.5 and 4.3 of the article. BH q, Benjamini–Hochberg adjusted p value within the exploratory family; it is reported for the two comparisons that reached significance before correction, since the adjusted values for the remaining comparisons do not affect interpretation (none approached significance).

Supplementary Table S4. Continuous Relationship Scales Questionnaire subscale analyses

Panel A. Pearson correlations (N = 314)

Variable	1	2	3	4	5
1. RSQ – Secure	1				
2. RSQ – Fearful	−0.161**	1			
3. RSQ – Preoccupied	−0.170**	0.146**	1		
4. RSQ – Dismissing	−0.269**	0.133*	0.127*	1	
5. Kerns Security Scale (child)	0.325**	−0.166**	−0.216**	−0.155**	1

* $p < 0.05$; ** $p < 0.01$. Subscales 1–4 are foster mother self-reports; variable 5 is the child's report.

Panel B. Multiple regression of children's attachment security on the four subscales (N = 314)

Predictor	r	β	SE	t	p	sr^2	VIF
RSQ – Secure	0.325	0.271	0.056	4.87	< 0.001	0.066	1.11
RSQ – Fearful	−0.166	−0.094	0.054	−1.74	0.083	0.008	1.05
RSQ – Preoccupied	−0.216	−0.150	0.054	−2.78	0.006	0.021	1.05
RSQ – Dismissing	−0.155	−0.051	0.055	−0.92	0.358	0.002	1.09

$R^2 = 0.144$, adjusted $R^2 = 0.133$, $F(4, 309) = 12.97$, $p < 0.001$. This table is reproduced as Table 9 in the article.

Panel C. Correlations corrected for attenuation

Subscale	Observed r with child security	Corrected r (α = 0.82)	Corrected r (α = 0.59)
RSQ – Secure	0.325	0.389	0.459
RSQ – Fearful	−0.166	−0.199	−0.234
RSQ – Preoccupied	−0.216	−0.259	−0.305
RSQ – Dismissing	−0.155	−0.186	−0.219

Corrections use $r \div \sqrt{(\alpha_x \times \alpha_y)}$ with $\alpha = 0.85$ for the Kerns Security Scale. Because internal consistency of the RSQ subscales ranged from $\alpha = 0.59$ to $\alpha = 0.82$ and the lowest value could apply to any subscale, corrected estimates are reported across the full range (section 3.4).

Supplementary File S1. Semi-structured interview schedule for foster mothers

The schedule was developed by the authors, reviewed by nine academic experts, and piloted with foster mothers not included in the main study (section 2.4.4). The Turkish original is given first, followed by an English translation. Prompts used by the interviewer are given in brackets.

A. Turkish original

1. "Koruyucu anne olmak" hakkındaki düşünce ve duygularınızı açıklar mısınız? (Koruyucu aile olmadan önceki ve koruyucu aile olduktan sonraki düşünce ve duyguları; ne hissettiniz?)

2. Koruyucu aile olma kararı ve sürecinde etkili olan içsel ve dışsal motivasyon kaynaklarınız nelerdi? (çocuk sahibi olma duygusu, çocuk özlemi, manevi duygular, çevrenin çocuk sahibi olma konusundaki ısrarcı soruları gibi)

3. Koruyucu ailesi olduğunuz çocuğunuzla ilk karşılaştığınızda neler hissettiniz, ilk karşılaşmanız nasıldı? Anlatır mısınız? (a) Siz neler hissettiniz, neler düşündünüz? (b) Nasıl davrandınız? (c) Çocuğunuzun davranışları nasıldı? [Alışma süreci nasıl oldu? Duygusal bir bağ kurma süreci nasıl oldu? İletişime geçme ve sürdürme konusunda sorun yaşandı mı?]

4. İlk günden bugüne siz ve çocuğunuz arasında ilişki kurma süreci nasıldı? [İlişkide değişiklikler oldu mu; bu değişiklikler hangi boyutta ve ne yönde oldu?]

5. Koruyucu ailesi olduğunuz çocuğunuzla "rutin/sıradan" bir gününüzü nasıl geçiriyorsunuz? Kısaca anlatabilir misiniz?

6. Çocuğunuz sizinle olan ilişkisi hakkında neler hissediyor, biliyor musunuz? [Bu hisleri nasıl ifade ediyor, nasıl gösteriyor? İlişkinizde en yoğun hangi duyguları yaşıyor?]

7. Koruyucu ailesi olduğunuz çocuk hangi durumlarda sizinle iletişim kurar? (üzüldüğünde, mutlu olduğunda, yalnız kaldığında, isteklerinin karşılanması sırasında) [Kendisinin aşamayacağı bir sorunla karşılaştığında ya da bir çatışma yaşadığında bunu sizinle nasıl paylaşır?]

8. Biyolojik anne olmayı ve koruyucu anne olmayı deneyimleyen birisi olarak her iki anneliğin size yaşattığı duyguları ve düşünceleri karşılaştırsanız neler söylersiniz?

9. "Çocuk" kelimesi sizin için ne anlama gelir? Açıklar mısınız?

10. Koruyucu aile olduktan sonra aile sisteminizde ne gibi değişiklikler oldu? Açıklar mısınız? (eş/partnerle ve varsa diğer çocuklarla olan yakın ilişkiler, biyolojik çocuk ile koruyucu çocuk arasındaki denge gibi)

B. English translation

1. Could you describe your thoughts and feelings about "being a foster mother"? (Thoughts and feelings before becoming a foster family and after; what did you feel?)

2. What were the intrinsic and extrinsic sources of motivation that shaped your decision to become a foster family and the process that followed? (for example the wish to have a child, longing for a child, spiritual feelings, persistent questions from those around you about having children)

3. What did you feel when you first met the child in your care, and what was that first meeting like? (a) What did you feel and think? (b) How did you behave? (c) How did the child behave? [How did the settling-in period go? How did an emotional bond develop? Were there difficulties in establishing and maintaining communication?]

4. How has the relationship between you and your child developed from the first day until now? [Have there been changes in the relationship; in what respects and in what direction?]

5. How do you spend a "routine/ordinary" day with the child in your care? Could you describe it briefly?

6. Do you know how your child feels about the relationship with you? [How does the child express or show these feelings? Which feelings are most present in your relationship?]

7. In which situations does the child in your care come to you? (when upset, when happy, when alone, when asking for something) [When the child meets a problem they cannot solve on their own, or has a conflict somewhere, how do they share it with you?]

8. As someone who has experienced both biological and foster motherhood, how would you compare the feelings and thoughts that each has given you?

9. What does the word “child” mean to you? Could you explain?

10. What changes occurred in your family system after you became a foster family? (for example close relationships with your spouse/partner and with your other children, if any; the balance between a biological child and a foster child)

Supplementary File S2. Semi-structured interview schedule for children

At the start of each child interview the interviewer asked the child how they addressed their foster mother and used the child’s own term throughout (section 2.4.4).

A. Turkish original

1. Bana biraz kendinden bahseder misin?

2. Koruyucu aile yanına yerleştirileceğini öğrendiğinde neler düşündün? Neler hissettin? Açıklar mısın?

3. Şimdi neler düşünüyor ve hissediyorsun? Anlatabilir misin?

4. Koruyucu anneni ilk gördüğünde, eve birlikte gittiğinizde neler hissettiğini açıklar mısın? [Koruyucu annenle geçirdiğin ilk gün senin için nasıldı? Neler hissettin, neler düşündün, neler yaptın?]

5. Koruyucu annenle geçirdiğin “normal” bir gününüzü kısaca anlatabilir misin? [Annenle ya da ailenin diğer fertleriyle ne kadar ve nasıl vakit geçirirsin?]

6. Koruyucu annenle olan ilişkini/iletişimini anlatır mısın? İlişkini nasıl tanımlarsın? [Zorlandığın durumlarda, çözemediğin olaylarda, üzüldüğün zamanlarda ilk kimin yanına gitmek istersin? Neden?]

7. Koruyucu anneni düşündüğünde aklına neler gelir? Anlatır mısın? [Koruyucu anneni bir şeye benzetmek istesen neye benzetirsin?]

B. English translation

1. Could you tell me a little about yourself?

2. What did you think when you learned that you would be placed with a foster family? What did you feel? Could you explain?

3. What do you think and feel now? Could you tell me about it?

4. Could you describe what you felt when you first saw your foster mother and when you went home together? [What was your first day with your foster mother like? What did you feel, what did you think, what did you do?]

5. Could you briefly describe an “ordinary” day with your foster mother? [How much time, and in what way, do you spend with your mother or with the other members of the family?]

6. Could you describe your relationship and communication with your foster mother? How would you define your relationship? [When you are struggling, when something happens that you cannot solve, when you are upset, who do you want to go to first? Why?]

7. What comes to mind when you think of your foster mother? Could you tell me? [If you were to compare your foster mother to something, what would it be?]

Supplementary File S3. COREQ 32-item checklist

Consolidated criteria for reporting qualitative research (Tong et al., 2007). Section numbers refer to the published article.

**Item**	**How it was addressed**	**Section**
Domain 1: Research team and reflexivity		
1. Interviewer/facilitator	All 20 interviews were conducted by the first author (CE).	2.6
2. Credentials	Social work academic; the study formed his doctoral research.	2.6; Acknowledgments
3. Occupation	University academic in social work; previously a social work specialist in the Ministry’s General Directorate of Child Services for seven years.	2.6; 2.7.2
4. Gender	Male.	2.6
5. Experience and training	Seven years of practice in the national child protection system; interviewing experience reported in the reflexivity statement. No separate formal qualitative-methods training is claimed.	2.7.2
6. Relationship established	No prior relationship with participants; access was arranged by caseworkers.	2.6
7. Participant knowledge of the interviewer	The interviewer introduced himself as a university researcher with no current Ministry role and no influence over placement decisions, and explained the purpose of the study.	2.6; 2.7.2
8. Interviewer characteristics	Positionality, assumptions and the risk of being read as an agent of the authority are stated in the reflexivity statement.	2.7.2
Domain 2: Study design		
9. Methodological orientation and theory	Explanatory sequential mixed-methods design; phenomenological approach to the interviews; reflexive thematic analysis; attachment transmission and secure base models as the interpretive framework.	1; 2.1; 2.7.2
10. Sampling	Purposive: foster mothers were selected so that all four RSQ attachment classifications were represented, and the child in each carer’s care was also interviewed.	2.3.2
11. Method of approach	Face to face and online, after access was arranged by the caseworkers of the provincial directorates.	2.3.2; 2.6
12. Sample size	10 foster mothers and the 10 children in their care (10 dyads, 20 interviews).	2.3.2

13. Non-participation	Numbers of families who declined were not recorded; foster mothers declined to be interviewed at home, so face-to-face interviews took place in the foster care units.	2.6; 4.9
14. Setting of data collection	Foster care units of the provincial directorates (7 dyads) and online (3 dyads).	2.6
15. Presence of non-participants	None. Foster mother and child were interviewed separately and in different locations; caseworkers were not present at any interview.	2.6
16. Description of sample	Age, education, employment, marital status, years fostering, service type, RSQ classification, and the corresponding child's characteristics.	2.3.2; Table 2
17. Interview guide	Two semi-structured schedules, reviewed by nine academic experts and piloted; provided in full.	2.4.4; Supplementary Files S1 and S2
18. Repeat interviews	No repeat interviews were conducted.	2.6
19. Audio/visual recording	Not permitted under the undertaking required by the Ministry; contemporaneous written notes were taken instead.	2.6; 4.9
20. Field notes	Contemporaneous written notes during each interview; analytic memos and an audit trail maintained in MAXQDA.	2.6; 2.7.2
21. Duration	Approximately 40 minutes with foster mothers and 25 minutes with children.	2.6
22. Data saturation	Not used. Sample size was determined by information power (Malterud et al., 2016).	2.3.2
23. Transcripts returned	No. Because recording was not permitted, passages that were incomplete or unclear were re-asked and clarified with the participant at the end of each interview.	2.6
Domain 3: Analysis and findings		
24. Number of data coders	One (the first author). The coding framework and a sample of coded material were independently reviewed by the second author and by an academic specialising in child protection.	2.7.2
25. Description of the coding tree	Codes were grouped into categories and then into themes and subthemes; a worked example is given, and the full structure is tabulated.	2.7.2; Tables 10 and 11
26. Derivation of themes	Themes were derived from the data; the carer's quantitative attachment classification was carried into the analysis as an attribute rather than as a coding frame.	2.7.2; 2.8
27. Software	MAXQDA 2024.	2.7.2
28. Participant checking	Participants did not comment on the findings. Clarification took place at the end of each interview.	2.6; 4.9
29. Quotations presented	Two excerpts per subtheme in the	3.6; 3.7; Supplementary File S4

	article, each attributed to a participant identifier with the carer's attachment classification; fuller quotations are provided.	
30. Data and findings consistent	Every interpretive claim is grounded in quoted material, and the two strands are set against each other in a joint display.	2.7.2; Table 3
31. Clarity of major themes	Four foster mother themes and three child themes, with their subthemes and principal categories.	3.6; 3.7; Tables 10 and 11
32. Clarity of minor themes	Divergent and negative cases are reported, including flat or avoidant relationships, conflict, self-reliance in place of help-seeking, and placement experienced as abandonment.	3.6; 3.7; 4.5

Supplementary File S4. Additional quotations from the foster mother and child interviews

The article gives two excerpts per subtheme. This file gives the fuller quotations from which those excerpts were taken, together with further quotations not used in the article. Identifiers follow the article (FM = foster mother, with the quantitative attachment classification; C = child). All quotations are near-verbatim contemporaneous records, since audio recording was not permitted (section 2.6). Because the child participants are under state protection, identifying details have been removed and particulars concerning health, family composition and the circumstances of individual children have been generalised; the wording of the quotations is otherwise unaltered.

Part 1. Foster mothers (section 3.6)

Theme 1: The process before becoming a foster mother (3.6.1)

Feelings and thoughts about foster motherhood.

> *Motherhood is not just about giving birth. Motherhood is not merely a title; it is the feeling of carrying responsibility for a child's life. While we set out to touch their lives, they touched ours; these children continue to grow with us, in our hearts. (FM5, secure)*

> *Being a foster family made me more sensitive and more timid. Of course, by the nature of the service model it contains fear as well, because the worry that the child could leave at any moment is dominant. (FM7, fearful)*

> *My only thought during the process was, what if I become very attached and they take my child from me, how would I live? And thoughts like, what if it does not work out, what if I cannot do it. There was the thought, might the child reject me? (FM3, preoccupied)*

Motivation for becoming a foster mother.

> *Because we did not have a child, I always longed for one. That longing was my strongest motivation. I wanted to take in a child, give them love, raise them with affection, and for them to know me as their mother, to embrace me, to love me in return. (FM7, fearful)*

> *It began when my neighbour took in a child. It saddened me to see children confined within four walls in an institution. Of course institutions provide care and do not leave children hungry, but children still*

need the love of a family. Everyone around me encouraged me and gave me the confidence to do it. (FM9, preoccupied)

The first encounter with the child.

Taking my child's psychology into account, in the first meeting I made eye contact rather than physical contact. Then I created a focus of interest around something she liked, and only slowly moved to closer contact. I asked permission, and I hugged her. When I hugged her she put her head on my chest. That was the day I felt I had become a mother. (FM5, secure)

We first met my foster child at the foster care unit. The staff there introduced us. Since it was an institutional setting and crowded, we could not sit for long. I cannot remember more than that. (FM3, preoccupied)

Theme 2: The foster motherhood process (3.6.2)

Building the relationship with the child.

We had no difficulty at all in the adjustment period. I sat and played with them, we shopped together, and they adjusted quickly. The first feeling of motherhood came the night they arrived, when I could not sleep and kept going into their rooms to check whether they were unwell or had kicked off their blankets. It felt as though they had always lived with me. Our home filled with peace. I realised how large my heart is, and how much my patience and my husband's have grown. (FM10, secure)

Emotionally I had many fears. Can I love him as much as his birth mother, can I fill that gap? And what if he does not call me mother, what if he does not want me. He was a traumatised child. There had been violence in his birth family, and whenever he saw a knife in my hand while I was cooking he would flinch. Because there had always been fighting at home, he had screaming fits and was frightened of loud noises. He reacted to everything. He resisted staying in a home environment. (FM7, fearful)

There were ups and downs with this daughter of mine. When she first came she cried a great deal, saying she missed her father. She was in intense distress in those first months and at one point tried to leave the house, saying she wanted to go back to the residential home. She received professional support around that time. (FM9, preoccupied)

Situations of interaction with the child.

Every morning we spend time in the kitchen while I prepare breakfast. When he comes home from school I sit with him while he does his homework. In the evenings we read together or watch a film he likes. Through this continuous interaction I see that the child feels safe and that the bond between us is getting stronger. (FM4, secure)

Then he watches some television, does his homework, goes to the park in front of the house, plays. (FM7, fearful)

During the week they go to school and come back and do their homework. Sometimes he talks to me and sometimes he does not. He sees his friends more often. (FM9, preoccupied)

Foster and biological motherhood from the foster mother's perspective.

I have tasted both kinds of motherhood. When I became a mother the second time it was no different from the first. My approach to each of my children is the same. (FM5, secure)

There is this difference in foster motherhood. You care for your biological children, you love them, that is normal. But here you love from the heart in a situation where you are not obliged to; you become attached afterwards. The small memories we accumulated all reinforce the love in my heart. (FM4, secure)

As a foster mother I did not see his birth, I could not witness his first moments. Not having lived those moments sometimes creates a feeling of deficiency in me, but every moment we spend together compensates for it. (FM8, secure)

Compared with my own biological motherhood I can say that my experiences with my foster child have been more demanding. Complaints come right to the door. We change classes, we change schools. (FM3, preoccupied)

The child's feelings in the relationship, as the foster mother perceives them.

They have a heartfelt way of calling out, my dear mother. That language of theirs bound me to them. They watch me for a long time without taking their eyes off me. If I am upset, their faces fall as well. (FM9, preoccupied)

My daughter has an intense fear of losing me. If she sees a separation scene on television her eyes fill immediately. She seems not to have got over her separation anxiety. (FM8, secure)

Theme 3: Meanings attributed to the concept of the child (3.6.3)

Child means the love of a son or daughter to me. It means sweetness, warmth, goodness, love and compassion. (FM9, preoccupied)

The word child is the code of life, I think. A person can learn a great deal from a child. A child is the only being in this life that is truly itself. (FM5, secure)

The word child means the joy of the house, its happiness. When the children were not here the walls closed in on me. The hours stopped, time did not pass. Now I do not know how the time goes. (FM10, secure)

Theme 4: The process after becoming a foster mother (3.6.4)

Our kinship relations are close-knit. Everyone welcomed the children warmly. My sister gave a large dinner for them. After the children came, happiness and liveliness increased in our family system. (FM10, secure)

My family were very supportive. They said, these children are a blessing for you, do not send them back. But my husband changed a great deal. His behaviour towards me used to be very restrictive; he did not want me to go out or to visit my mother. After we took the children he himself says, go out, take a walk, so that you do not feel closed in. He suggested I get a driving licence to make things easier with the children. (FM9, preoccupied)

When we had our biological child we called him our second child, because our first child was our foster child. The siblings were very respectful towards each other. (FM5, secure)

Part 2. Children (section 3.7)

Theme 1: The child's relationship with the foster mother (3.7.1)

Strong bonds and belonging.

My foster mother and father are like my real family; they never made me feel like an outsider. I do not see my mother as a foster mother. There are older children in my foster family and I am much loved. We go shopping together, we go on picnics, we visit relatives. My relationship with my mother in particular is very strong. (C2)

They are not like my birth family, but they are far better in many ways. I feel closer to them. Actually I feel as though my mother gave birth to me. When I close my eyes and think of the word mother, it is her I see. (C5)

Functionality of the relationship.

I never went without a mother while I was with her. She is glad when she hears good news, she supports me when I am upset. When I do something wrong she tries to support me. (C1)

We chat, we talk, she listens to me constantly. When I have any trouble I can share it with my mother without hesitation; in fact my mother usually understands me from my face. (C2)

Non-dynamic relationships.

With my grandmother we are neither very attached nor very separate. She talks with me, but then continues as though she were talking to any ordinary person. Recently I would say there has been a decrease in the things she does for me. (C1)

With my mother we are sometimes good and sometimes bad, in short. How shall I put it, I am watching television and she says do not watch. When I am doing something while she cleans the house she immediately says do not do that, and when I ask why not, we fall out and it becomes bad. (C3)

When I am struggling, when something happens that I cannot solve, I do not want to go to anyone. Because I pull myself together on my own, I do not want to go to anyone. (C9)

Theme 2: The process before placement with the foster mother (3.7.2)

A sense of family.

Because I was three I do not really remember, but I felt as though I had always been with them. When I first went home with my foster mother the family were waiting for us; it was a beautiful day. I have photographs from that time and I feel very happy when I see them. One of the moments I can never forget was when I first saw my foster mother, and when my hesitation went, I felt a rush of joy. (C2)

A welcome surprise had been prepared at home. Before I arrived they had cooked a meal, prepared my room and bought clothes for me. I describe my relationship with my foster mother as a real mother-and-daughter relationship. (C8)

Uncertainty and anxiety.

When I learned I would leave the institution and go to a family, I was excited, I hoped a good family would come. But I also thought, will it be a good family or a bad family. When I first saw my foster mother I was afraid at first, I looked to see what was happening. (C7)

I remember the room I was given when I had only just arrived, at four. I was not crying then, I was thinking, what am I going to do here. I did not even know that I had been fostered. (C3)

Feeling safe.

I already knew my mother beforehand. When I learned I would go to her I was happier. When I went to my mother's house it was a completely different feeling; I was going to a home for the first time. I was very happy, I was very excited. (C5)

Abandonment and loneliness.

At first I was very upset because my own family left me and went. I did not want to come here at all, but they left me here by force. And I tried to get myself used to living in this family. (C10)

Theme 3: The perceived metaphor of foster motherhood (3.7.3)

Safe harbour.

A mother is someone with whom you share your troubles and pour out your heart. It means sacrifice, solving problems, trust. (C2)

When I think of my foster mother, love and great happiness come to mind. I compare my foster mother to family, and family is a beautiful thing. (C10)

Guide and light.

My mother is more like a preschool teacher to me. She helps me with things I do not know; she is both my mother and my teacher. She is like a light that illuminates my path. (C4)

Protective shield.

I would compare her to an angel and to a heart. Whenever I am hurt I feel my mother beside me. (C8)

My foster mother always seems like a guardian angel to me. When she is beside me I feel safe. Her love and support make me feel as though I am growing wings. (C4)

Loving from the heart.

When I was seven I questioned this a lot: is a child born from the heart or from the belly? My mother said, from the heart. I did not understand it then. Now I understand. (C3)
